# Substrate Specificity within a Family of Outer Membrane Carboxylate Channels

**DOI:** 10.1371/journal.pbio.1001242

**Published:** 2012-01-17

**Authors:** Elif Eren, Jagamya Vijayaraghavan, Jiaming Liu, Belete R. Cheneke, Debra S. Touw, Bryan W. Lepore, Mridhu Indic, Liviu Movileanu, Bert van den Berg

**Affiliations:** 1 University of Massachusetts Medical School, Program in Molecular Medicine, Worcester, Massachusetts, United States of America; 2 Syracuse University, Department of Physics, Syracuse, New York, United States of America; University of Zurich, Switzerland

## Abstract

Many Gram-negative bacteria, including human pathogens such as *Pseudomonas aeruginosa*, do not have large-channel porins. This results in an outer membrane (OM) that is highly impermeable to small polar molecules, making the bacteria intrinsically resistant towards many antibiotics. In such microorganisms, the majority of small molecules are taken up by members of the OprD outer membrane protein family. Here we show that OprD channels require a carboxyl group in the substrate for efficient transport, and based on this we have renamed the family Occ, for outer membrane carboxylate channels. We further show that Occ channels can be divided into two subfamilies, based on their very different substrate specificities. Our results rationalize how certain bacteria can efficiently take up a variety of substrates under nutrient-poor conditions without compromising membrane permeability. In addition, they explain how channel inactivation in response to antibiotics can cause resistance but does not lead to decreased fitness.

## Abstract 2

Characterization of a large family of outer membrane channels from gram-negative bacteria suggest how they can thrive in nutrient-poor environments and how channel inactivation can contribute to antibiotic resistance.

## Author Summary

Emerging antibiotic resistance in the treatment of infectious disease is an increasing problem that urgently requires new drug development. In Gram-negative bacteria, the outer membrane (OM) prevents permeation of small molecules, including antibiotics. A family of channel-forming proteins, called OprD proteins, are present in the OM to enable uptake of nutrients required for growth and cellular function. Since these channels also transport antibiotics, understanding how molecules are recognized and transported by these proteins should enable the design of more effective antibiotics. Here, we have characterized by biophysical and biochemical methods the structures and substrate-specificities of nine members of the OprD channel family of a common multidrug-resistant pathogen, *Pseudomonas aeruginosa*. Because we demonstrate that efficient passage through these channels requires the presence of a carboxyl group in the substrate, we renamed this channel family outer membrane carboxylate channels, or Occ. This broad substrate specificity suggests that such efficient transport allows bacteria to thrive in nutrient-poor environments. We also show markedly varied substrate specificities among the family members, especially for antibiotics, suggesting that mutation of a single channel can result in antibiotic resistance. These results provide the framework for studying the interaction of antibiotics with OM uptake channels, which will facilitate the development of more permeable and thus effective drugs.

## Introduction

To acquire water-soluble, low-molecular-weight compounds that are required for cell growth and function, Gram-negative bacteria contain uptake channels within the outer membrane (OM). These channels mediate uptake by passive diffusion along a concentration gradient that is present across the OM [1]. Two classes of channels can be distinguished, which differ in the way they interact with substrates. The non-specific channels or porins (e.g., *E. coli* OmpF and OmpC) do not bind their substrates with measurable affinities [1],[2]. Hence, such channels are thought to be most effective when the external substrate concentrations are high. Moreover, porins have rather large pores, allowing the passage of any polar compound below a certain size limit (∼600 Da for OmpC/F), including many antibiotics.

The other class of OM diffusion channels consists of substrate-specific channels. As the name implies, these channels have binding sites for their substrates and are therefore substrate specific. Substrate-specific channels occur in all Gram-negative bacteria and are dedicated to the uptake of different classes of substrates: examples in *E. coli* are the sugar channel LamB [3],[4] and the nucleoside channel Tsx [5]–[7]. Functionally, the binding of substrates by these channels may render them effective when the external substrate concentrations are low. Hence, they are especially prevalent in bacteria that thrive in nutrient-poor environments, such as pseudomonads. In fact, pseudomonads and related bacteria do not have large-channel porins and rely on substrate-specific channels for the acquisition of small water-soluble compounds. As a consequence, the OM of such bacteria is approximately two orders of magnitude less permeable than that of porin-containing bacteria such as *E. coli* [8]. The intrinsic low permeability of the OM poses an obvious and severe problem for the treatment of infections mediated by such organisms, most notably *Pseudomonas aeruginosa* and *Acinetobacter baumannii*. These organisms are notoriously resistant to many antibiotics and are responsible for many hospital-acquired infections within the United States [9]–[11]. The intrinsic antibiotic resistance can be compounded by acquired resistance, which includes inactivation of OM channels that mediate antibiotics uptake.

*P. aeruginosa* has substrate-specific OM channels dedicated to the uptake of, e.g., phosphate (OprP; [12]) and sugars (OprB; [13],[14]). However, most of the water-soluble small molecules are thought to be taken up by members of the Outer membrane carboxylic acid channel (Occ) family (formerly OprD family; see Text S1 for Supporting Discussion and 1). This family is notable for the presence of many members in pseudomonads and related bacteria [15]. The 19 family members present in *P. aeruginosa* and the five family members in *A. baumannii* have 35%–45% pairwise identity. Phylogenetic analysis suggests a division into OccD (formerly OprD) and OccK (formerly OpdK) subfamilies [16]. The OccD1 (OprD) channel is the archetype of the entire family and is thought to be a channel for basic amino acids [17],[18]. In addition, OccD1 has attracted much interest since it appears to serve as the entry portal for carbapenem β-lactam antibiotics [19],[20], one of the few classes of antibiotics that are effective against *P. aeruginosa*. Based on indirect approaches, putative substrate specificities of several additional OccD and OccK subfamily members have been proposed [16],[18],[21],[22]. Experimental evidence for this is, however, lacking.

X-ray crystal structures of OccD1 and OccK1 have previously been determined at medium resolution [23],[24]. Both proteins form monomeric 18-stranded barrels with a central channel that is constricted by two extracellular loops. A striking feature in both structures is the presence of a “basic ladder,” a row of arginine and lysine residues that leads to and away from the constriction, and which was hypothesized to interact with a substrate carboxyl group [23]–[25]. These structures allowed us to begin to understand the transport mechanism of Occ family members and the basis for their putative substrate specificity.

In order to elucidate the substrate preferences and putative specificities of Occ channels, we report here the characterization of a number of Occ channels by a multidisciplinary approach involving X-ray crystallography, electrophysiology, and in vitro transport experiments. Together, the data establish that the Occ family consists of carboxylate transport channels with markedly varied substrate specificities, especially for antibiotics. Our results not only provide insight into bacterial physiology, but also form a starting point for understanding the interactions of antibiotics with *P. aeruginosa* OM channels, which should lead to the rational design of novel compounds with superior permeation properties.

## Results

### Crystal Structures of Occ Family Members Show Large Variations in Pore Sizes

**Figure 1:**
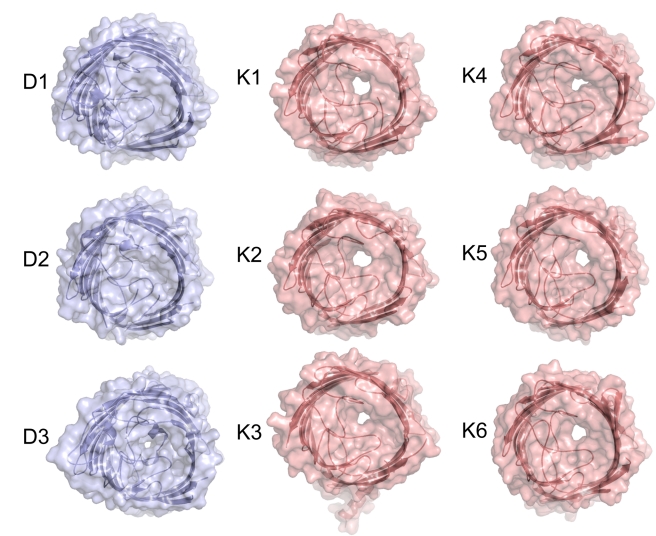
Crystal structures of Occ channels show a wide variety in pore sizes. Transparent surface representations from the extracellular side for OccD1–D3 (blue) and OccK1–K6 (salmon). The channels are shown in identical orientations. This and other structure figures were made with PYMOL (The PyMOL Molecular Graphics System, Schrödinger, LLC).

To determine if the phylogenetic subdivision [16] of Occ proteins exists at the structural level, we determined X-ray crystal structures for seven Occ family members (OccD2, OccD3, and OccK2–K6; Materials and Methods and Table S2). In addition to these new structures, we obtained much higher resolution data and more complete structures for OccD1 (formerly OprD; 2.15 Å versus 2.9 Å previously) and OccK1 (formerly OpdK; 1.65 Å versus 2.8 Å previously). All channels crystallize as monomers. As expected from pairwise sequence identities that range from 35%–50%, the Occ proteins share a similar architecture, with a slightly off-center constriction formed by the inward-folded extracellular loops L3 and L7 and residues in the barrel wall (Figure 1) [23],[24]. A notable exception to the constriction architecture is OccD3 (formerly OpdP). This channel has an N-terminal extension of 25–30 residues (Figure S1), and this extension is responsible for constricting an otherwise large-diameter pore within the crystal structure (Figure S2).

A comparison of the crystal structures suggests that the division into the OccD and OccK subfamilies occurs on a structural level as well; the OccD subfamily members of which structures were determined have much smaller pores than those of the OccK subfamily. In fact, OccD2 (formerly OpdC) is closed in the crystal structure. OccD1 does have a very small pore, which is not visible in the orientation of Figure 1 since it is obstructed by part of an approximately eight-residue-long insertion in loop L7 (Arg287-Gly294; Figure S1 and Figure S2). This L7 insertion is present only in a few OccD subfamily members and absent in the OccK subfamily (Figure S1). An abundance of small residues (^287^RNGSGAGG^294^ in OccD1) makes the L7 segment likely to be flexible. Indeed, residues 287–292 were not visible in the original crystal structure of OccD1 (PDB ID: 2ODJ), and the remainder had a different conformation compared to the current structure. As a result, the new OccD1 structure has a smaller pore than previously observed (Figure S2).

The pore sizes of all OccK subfamily members for which structures were determined are substantially larger than those of the OccD1–3 channels (Figure 1), suggesting that the OccD and OccK subfamilies have different substrate specificities. As a comparison, the OccK1 pore is slightly larger in diameter than that of *E. coli* LamB, which is a channel specific for sugars [3]. The pores of the proteins within the OccK subfamily show a considerable variation in size and shape, suggesting differences in substrate preferences within subfamilies as well (Figure 1 and Figure S3). This notion is borne out by the fact that the sequence conservation among the residues that line the pore constriction is very low, with the exception of certain basic ladder residues (Figure S1).

### Conservation of the Basic Ladder in Occ Family Members

**Figure 2:**
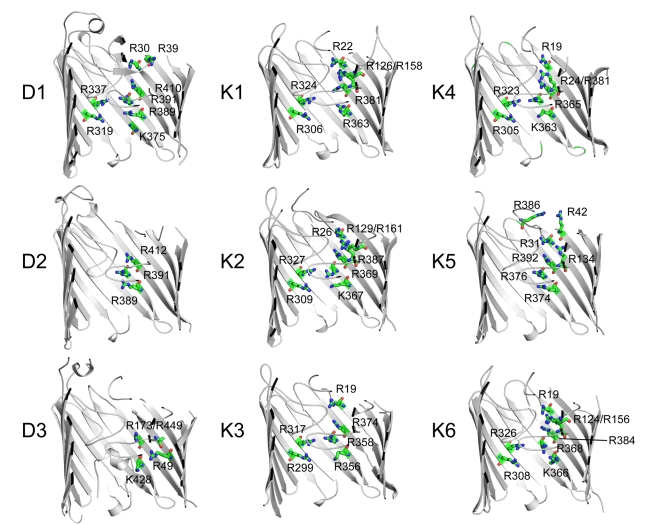
Conservation of the basic ladder in Occ channels. Stick models of the arginine and lysine residues that form the basic ladder in Occ channels with solved crystal structures. Residues are numbered. Channels are shown in identical orientations.

A prominent structural feature that was identified in the original structures of OccD1 and OccK1 is the basic ladder, a row of arginine and lysine residues in the barrel wall. The side chains of these residues point into the lumen of the barrel and were proposed to form an electrophoretic conduit that would bind substrate carboxylate groups to guide the substrates towards the constriction [23],[24]. The structural data now show that the basic ladders are likely present in all Occ channels (Figure 2 and Figure S1). In most family members, 6–8 residues contribute to the ladder, but in some channels only the three central ladder residues closest to the constriction are present (Arg389, Arg391, and Arg410 in OccD1), suggesting that the other basic residues may be dispensable. Thus, the basic ladder is conserved in Occ proteins, an observation which strongly suggests that this structural feature is important for substrate selectivity. Interestingly, the closed state of the OccD2 channel observed in the crystal structure features a strong interaction between the carboxyl group of Asp294 and the side chain of the central basic ladder residue Arg389. We speculate that the carboxylate in this interaction mimics that of the substrate during transport.

### Electrophysiology Data Show That Occ Channels Are Dynamic

**Figure 3:**
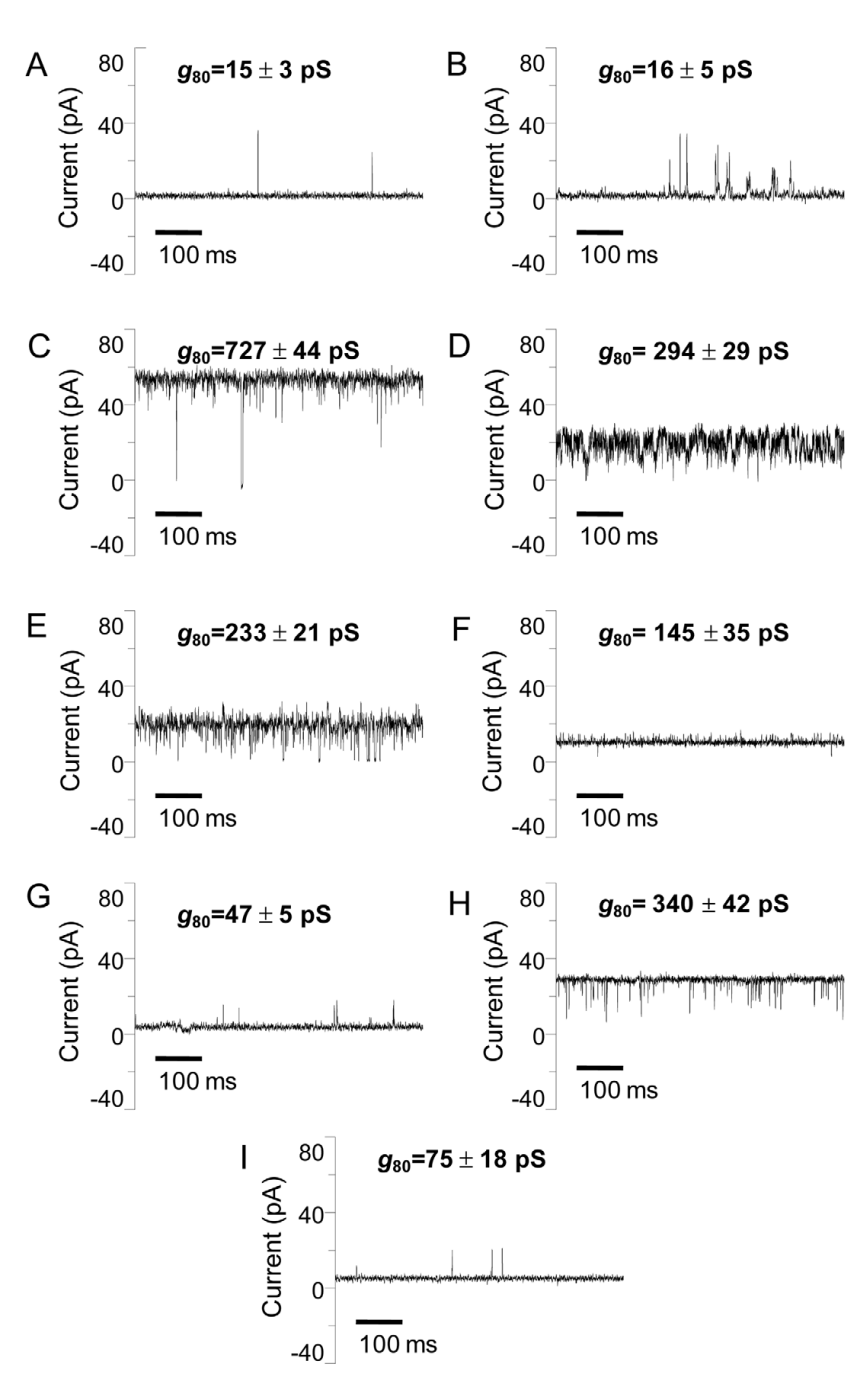
Representative single-channel electrical recordings of the members of the OccD and OccK subfamilies with solved crystal structures. (A) OccD1; (B) OccD2; (C) OccD3; (D) OccK1; (E) OccK2; (F) OccK3; (G) OccK4; (H) OccK5; and (I) OccK6. The data were collected at an applied transmembrane potential of +80 mV. The buffer solution in the chamber contained 1 M KCl, 10 mM potassium phosphate, pH = 7.4. For the sake of clarity, the single-channel electrical traces were low-pass Bessel filtered at 2 kHz. The numbers located above the traces represent the single-channel conductance of the most probable substate of the channel. The averages were derived from at least three independent single-channel electrical recordings.

To complement the crystal structures with time-resolved information of the channels in native-like environments, we recorded single-channel planar lipid bilayer data for all over-expressed Occ proteins (Figure 3 and Figure S4). The observed traces are generally consistent with the available crystal structures. For example, the dominant states of OccD1 and OccD2 have very small conductance values (∼15 pS), in accordance with the small/closed pores observed in the crystal structures (Figure 1). Interestingly, both channels show occasional upward spikes in conductance, suggesting the occurrence of infrequent gating events that form a larger pore. Remarkably, the conductance of the dominant state of OccD3 is the largest of all channels analyzed (∼700 pS), which is clearly not compatible with the small pore observed in the crystal structure of this protein (Figure 1). However, a displacement of the N-terminal segment caused by the conditions in the electrophysiological experiments (high ionic strength, applied voltage) would generate a large-diameter pore (Figure S2). OccD3 does show an infrequent state characterized by a very low conductance (Figure 3C), which could correspond to a pore where the N-terminus forms part of the constriction, as observed in the crystal structure.

Interestingly, the remaining members of the OccD subfamily that were characterized (OccD4–D6) show dominant states with conductance values ranging from very low (OccD5) to relatively large (OccD6) comparable to that measured previously for OccK1 (Figure S4). Thus, the single-channel data suggest that the crystal structure-derived division into OccD and OccK subfamilies based on pore sizes is too simplistic, and that some OccD proteins may have similar-sized pores as OccK proteins. All channels exhibit states that are characterized by very small conductance values and that are populated to different extents. Thus, the single-channel data suggest that OccD subfamily members have dynamic channels that are characterized by several states that range from virtually closed to having sizeable openings. The observed gating is unusual, but not unprecedented for diffusion-driven channels [26],[27]. The ability to observe different states of the same channel is a clear advantage of electrophysiology, complementing the static high-resolution information from X-ray crystal structures.

For OccK channels, conductance values are on average somewhat larger than those of OccD subfamily members, ranging from ∼50 pS for OccK4 to ∼300 pS for OccK1 [23],[28], OccK5, and OccK7. These data are consistent with the fairly large pores observed in the six crystal structures of members of this subfamily. Generally speaking, the observed conductance values are in the same range as that of other substrate-specific channels recorded under similar conditions (e.g., LamB monomer, ∼155 pS [29]), but considerably lower than those of general, non-specific porins (e.g., OmpF monomer, ∼700 pS [30]). Finally, the conductance data show that most OccK channels are dynamic and characterized by several substates as well (Figure 3).

### In Vitro Transport Data Show That Occ Channels Are Substrate Specific

We sought to define the substrate specificities of a number of Occ proteins to answer the question of whether, and to which extent, these channels are substrate specific. To do this we investigated several in vitro systems. We performed two types of experiments: (i) “direct” uptake assays using radiolabeled substrates and (ii) measurement of radiolabeled substrate uptake in the presence of excess amounts of unlabeled compounds. In principle, the latter experiments only provide information about substrate binding to the channel constriction and not necessarily about transport. However, the absence of binding in such experiments is a strong indication that a compound is not a good transport substrate. Moreover, due to the large number of required radiolabeled compounds, a comprehensive definition of substrate specificity by direct transport assays alone is not practical.

We initially performed uptake measurements using proteoliposomes with purified OccD1 and ^3^H-arginine as substrate. However, even after extensive optimization efforts we could only obtain uptake levels that were ∼5-fold of that of empty liposomes, even when a substrate-binding protein was included in the proteoliposome lumen (Figure S5). We next developed a system in which we overexpressed Occ channels on an inducible plasmid in the porin-deficient *E. coli* strain Bl21 omp8, followed by the production of total membrane vesicles. Using this system we could obtain transport activities for a number of substrates of up to 50-fold of that of the background vesicles. Since the levels of the proteins within the vesicles are not identical, we have expressed substrate uptake as specific activities, i.e., the uptake activities are corrected for the different amounts of Occ channels in our vesicles (Figure S6). The observed uptake levels are very high for diffusion-based transport and are probably caused by a non-random, “right-side-out” orientation of the channels within the vesicles (Figure S7). Importantly, the results are produced using physiologically relevant, very low substrate concentrations (sub-µM for most substrates; Figure S8).

We based our choice of substrates on the predictions made by Hancock et al. [16] and on their availability in radiolabeled form. We tested arginine (potential substrate for OccD1), benzoate (various OccK family members), vanillate (OccK1), glucuronate (OccK2), pyroglutamate (OccK3), phenylacetate (OccK4), and citrate (OccK5). We also tested glucose as a substrate due to the general importance of sugars as carbon sources. Substrate concentrations (Figure S8) and uptake times (Figure S9) were determined for each individual substrate using one channel with good activity; these conditions were subsequently used for the entire panel of channels. In addition to 13 Occ channels we also tested *E. coli* OmpG and *E. coli* FadL. OmpG is a monomeric porin of unknown function with a large pore that likely forms a non-specific channel (positive control). FadL is a specific channel for the uptake of long-chain fatty acids (negative control). As an additional positive control we used vesicles made from C43 (DE3) cells, which contain all the OM proteins that are lacking in omp8 cells (i.e., OmpF, OmpC, OmpA, and LamB).

**Figure 4:**
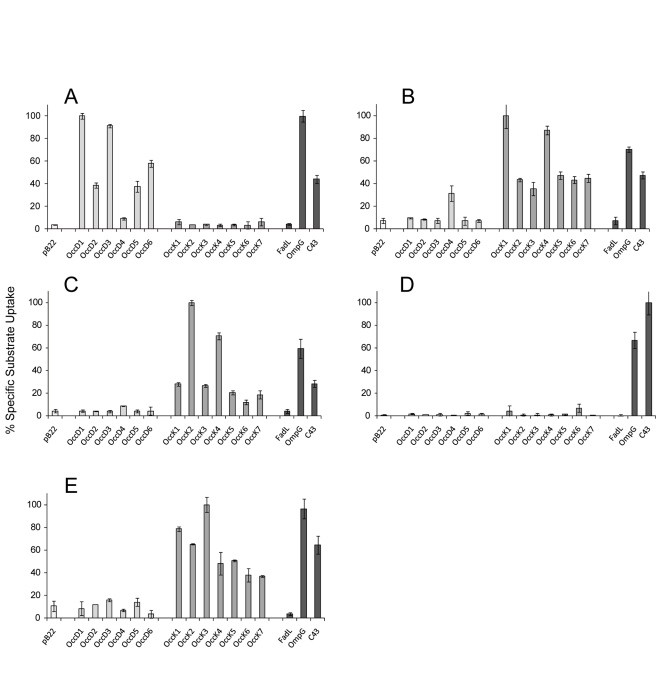
In vitro transport assays show a division of Occ channels into two subfamilies. Uptake of radiolabeled substrates in total membrane vesicles of *E. coli* Bl21 omp8 expressing empty plasmid (pB22), Occ channels, and *E. coli* OmpG or FadL. In addition, uptake mediated by the porin-containing strain C43 (DE3) is shown. Substrates are (A) arginine (0.25 µM, 15 min uptake), (B) benzoate (0.5 µM, 10 min), (C) glucuronate (0.5 µM, 10 min), (D) glucose (0.5 µM, 15 min), and (E) pyroglutamate (0.5 µM, 15 min). 100% specific activities correspond to 199.7±5.5 (A), 40.4±0.7 (B), 49.7±1.1 (C), 36.9±1.4 (D), and 20.7±0.5 (E) pmoles substrate/min/mg protein. Filter backgrounds are subtracted from all measurements.

The uptake experiments for arginine are striking in that they show a clear functional distinction between both Occ subfamilies (Figure 4A). All OccD channels, with the exception of OccD4, show good levels of arginine uptake. By contrast, the OccK channels have arginine transport activities that are around background levels. Thus, good substrates for OccD channels are poor substrates for OccK channels. The efficient arginine uptake mediated by OccD3 is noteworthy since this protein was shown, like OccD1, to mediate arginine uptake in *P. aeruginosa* in vivo [18]. Since several OccD channels besides OccD1 and OccD3 mediate arginine uptake, our data also explain the observation that an *occD1/occD3* double knockout still grows in the presence of arginine as a sole carbon source [18].

We next asked whether putative OccK substrates, such as aromatic acids [16],[31], are transported by OccD family members. First, we tested benzoate. This compound is indeed a good substrate for OccK proteins and a poor substrate for OccD family members (Figure 4B). The most efficient benzoate uptake was observed for OccK1, which correlates well with the prediction of this protein as a vanillate channel [16] and with its high structural similarity to the putative benzoate channel BenF from *Pseudomonas fluorescens* [31]. Vanillate (Figure S10), while being transported by OccK1, is clearly not the preferred substrate for this channel since relatively high substrate concentrations (10 µM) and long uptake times had to be used to obtain relatively low specific activities (Figure S8 and Figure S11). This result is consistent with previous single-channel experiments, where the affinity of vanillate for OccK1 was found to be quite low (∼2 mM) [23]. Moreover, extensive soaking and co-crystallization experiments of vanillate with OccK1 have not been successful. Notably, while the current OccK1 structure contains two vanillate molecules, they are not bound at positions relevant for transport (Figure S12), confirming that vanillate is not a good substrate for OccK1. The final aromatic acid tested was phenylacetic acid, proposed to be a substrate for OccK4 and OccK11 [16]. OccK4 was indeed the most efficient phenylacetate uptake channel of the proteins tested; however, the specific activities are very low and, as for vanillate, high substrate concentrations had to be used (Figure S8 and Figure S11). Thus, phenylacetate is not a preferred substrate for any of the tested channels.

We next assayed the substrate couple glucuronate/glucose, to test the importance of the presence of a carboxyl group in the substrate on transport (Figure S10). Like benzoate, glucuronate is a good substrate for OccK channels but not for OccD subfamily members (Figure 4C). The highest transport rates are observed for OccK2, which agrees well with previous predictions [16]. It should be noted that glucuronate is not transported by OccD1 in our assay, a finding that contrasts with previous results obtained in growth experiments with high (mM) substrate concentrations [21]. Strikingly, glucose, as is evident from the low specific activities, is a poor substrate for all Occ channels tested, with the exception of low uptake levels mediated by OccK6 (Figure 4D). The glucuronate/glucose results show that the presence of a carboxyl group in the substrate is required for efficient transport by Occ channels.

The next substrate tested, pyroglutamate, has a similar activity profile compared to benzoate/glucuronate (Figure 4E). OccD proteins display transport levels that are similar to background, whereas OccK subfamily members are much more efficient. As with benzoate/glucuronate, the channel that is most efficient in pyroglutamate uptake (OccK3) was indeed predicted to transport this and related five-membered ring compounds [16].

Citrate, the final substrate tested, is representative of a large group of organic acids that are important carbon sources in vivo. Moreover, tricarboxylic acids were predicted to be substrates for the OccK5 channel (formerly OpdH) [16]. Surprisingly, however, citrate is a very poor substrate for all Occ channels including OccK5 (Figure S8 and Figure S11), suggesting that the presence of multiple carboxylates in a substrate may be detrimental to transport. Previous electrophysiology experiments support our results, as no citrate binding to OccK5 could be detected [22].

**Figure 5:**
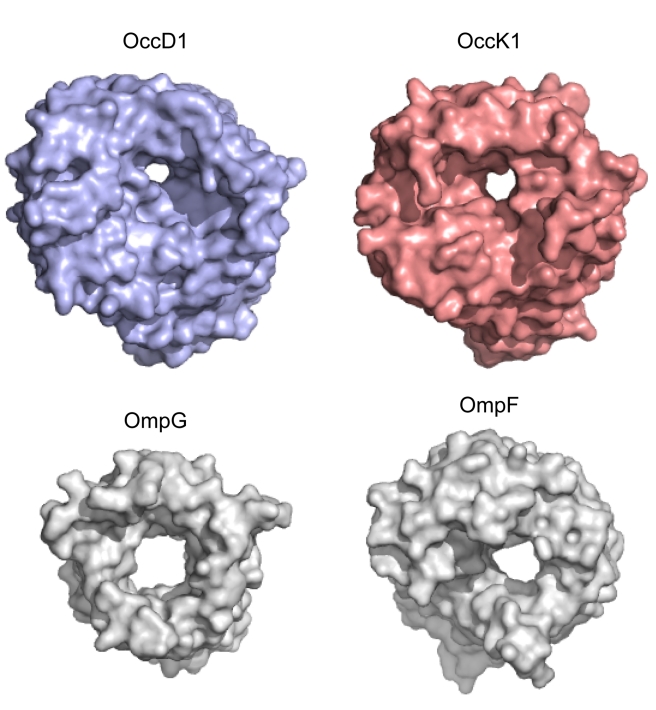
Small substrate-specific pores can mediate efficient substrate uptake. Comparison of pore sizes of OccD1 (PDB ID: 2 ODJ) and OccK1 with those of the non-specific porins OmpG and OmpF. The surface views are shown from the extracellular side.

The control channels OmpG and FadL behave as expected in our transport assays, with generally efficient substrate uptake (including glucose) mediated by OmpG and no uptake observed for FadL. Efficient substrate uptake is also observed with the OmpF/OmpC porin-containing strain C43 (DE3). An important picture that emerges from these comparative data is the high efficiency of Occ channel-mediated uptake at low substrate concentrations. In many cases, Occ channels are as effective as or more effective than OmpG and OmpF/C, despite the latter proteins having much larger pores than the Occ family members (Figure 5). These observations demonstrate that substrate-specific channels, by binding their substrates, function efficiently when substrate concentrations are low.

### Definition of Occ Channel Substrate Specificity

Next, we set out to define the substrate specificities of Occ channels in more detail. The uptake experiments with radiolabeled substrates described in the previous section are not practical for a more comprehensive definition of substrate preferences. Instead, we have defined the substrate specificities of OccD1, OccK1, and OccK2 by monitoring transport of radiolabeled substrates in the presence of a 10-fold excess of an unlabeled compound. If that compound is a transport substrate, it will compete with the labeled substrate for binding to the channel, resulting in lower transport levels of the substrate. The main advantage of this method is that a relatively large number of compounds can be screened rapidly. The results of these experiments are shown in Table 1. The data for OccD1 are striking in that they show that the substrate specificity of this channel is quite narrow. The only compounds that lower arginine uptake when added in a 10-fold excess are basic amino acids. Agmatine affects arginine uptake only slightly, confirming the need for a carboxyl group in the substrate. The Arg-Arg dipeptide is also effective in competing with arginine uptake, suggesting that peptides containing basic residues are also substrates of OccD1. D-arginine also affects arginine uptake, but clearly not as efficiently as the L-amino acid. The other amino acids tested did not show any competition, even at a 100-fold molar excess (Table S3). Likewise, no effect on arginine uptake was observed for sugars, aromatic compounds, and organic acids. Thus, OccD1 is a very specific channel for basic amino acid uptake.

**Table 1:** Definition of Occ channel specificity.

	OccD1	OccK1	OccK2
Control	100^a^±9	100±8	100±8
**Amino acids**			
*Lysine*/*Arginine*	**3±1** b/**2±1**	100±4/88±3	86±2/90±4
*D-arginine*/*Histidine*	**49±1**/**12±2**	94±3/100±4	92±5/83±4
*Ornithine*/Agmatine	**1±2**/83±2	92±4/100±1	96±6/100±6
*Aspartic Acid*/*Proline*	98±1/97±4	55±9/79±2	66±4/66±3
*Leucine*/*Tyrosine*	99±3/96±4	87±6/80±7	100±6/89±7
*Serine*/*Pyroglutamate*	95±8/89±3	62±7/**15±1**	100±9/**25±2**
*Glutamic Acid*	99±2	85±3	59±9
*Arg-Arg dipeptide*	**40±1**	89±5	91±2
**Sugars**			
Glucose/Sucrose	100±5/99±2	89±3/93±2	100±6/85±5
Galactose/Arabinose	86±5/95±1	96±4/85±3	74±6/88±4
*Gluconate*/*Glucuronate*	100±3/97±6	80±8/**20±6**	100±3/**3±3**
**Aromatic compounds**			
*Benzoate*/*Vanillate*	98±2/95±2	**1+3**/**37±3**	**20±6**/81±5
*3-nitrobenzoate*	100±6	**29±4**	55±5
*4-nitrobenzoate*	100±6	**16±3**	**48±4**
**Organic acids**			
*Lactate*/*EDTA*	65±10/97±1	99±8/84±3	100±8/100±6
*Citrate*/*Cis-aconitate*	99±3/100±5	100±6/99±6	100±5/100±5
*Succinate*/*Malonate*	100±6/100±5	100±8/100±6	94±5/92±2
*Tartrate*/*Adipate*	97±3/100±2	100±5/**38±3**	100±5/100±3
**Fatty acids/alcohols**			
*Caproate*/*Octanoate*	100±6/98±3	**20±5**/**33±6**	99±4/100±3
Octanol	91±7	95±3	100±6
**Others**			
Adenosine/Thymidine	96±8/98±9	100±5/99±5	70±3/91±4
Indole/Imidazole	99±2/85±5	100±7/100±3	100±8/98±3

Uptake of radiolabeled arginine by OccD1, benzoate by OccK1, and glucuronate by OccK2 was measured in the presence of a 10-fold excess of unlabeled low-molecular weight compounds. In all cases, total levels of uptake are reported, expressed as a percentage of uptake in the absence of unlabeled compound (100%). Substrates containing a carboxyl group are italicized.

a Reported values are the average of two or three experiments.

b Efficient inhibition values, defined as resulting in <50% transport, are shown in bold.

Completely different profiles are observed for OccK1 and OccK2. When present in 10-fold excess, amino acids do not affect transport by these channels, with the sole exception of the cyclic amino acid derivative pyroglutamate, which lowers transport mediated by both channels effectively (Table 1). The only sugar that is effective is glucuronate, which, together with gluconate, is the only tested sugar with a carboxyl group. The fact that gluconate does not compete with transport suggests that OccK1 and OccK2 prefer cyclic carboxyl-containing molecules as substrates. Consistent with this notion, the aromatic compounds are generally effective in lowering transport, especially for OccK1. Importantly, as judged from the substrates for which both transport and competition data are available, the competition profiles correlate well with transport, i.e., compounds that are efficient competitors are also transported well, and *vice versa* (e.g., pyroglutamate and OccK1 and OccK2; Figure 4 and Table 1). Thus, the use of a competition assay to assess transport appears to be justified. No effect is observed for the organic acids, with the exception of the dicarboxilic acid adipate, which lowers OccK1-mediated transport fairly efficiently. An interesting finding is the efficient lowering of OccK1-mediated benzoate transport by octanoate and caproate (Table 1), suggesting that OccK1, but not OccD1 and OccK2, can serve as an uptake channel for medium-chain and possibly short-chain fatty acids. OccK1-mediated transport is not affected by octanol, again illustrating the importance of a carboxyl group in the substrate. In conclusion, the substrate specificities of OccK1 and OccK2 are not as narrow as that of OccD1, perhaps due to their larger pores. Various monocyclic compounds are efficient substrates for these channels, provided that they possess a carboxyl group. OccK1 is the least specific channel, also allowing the efficient passage of certain linear compounds such as medium-chain fatty acids.

### Occ Channels Show High Specificity for Antibiotics Uptake

**Figure 6:**
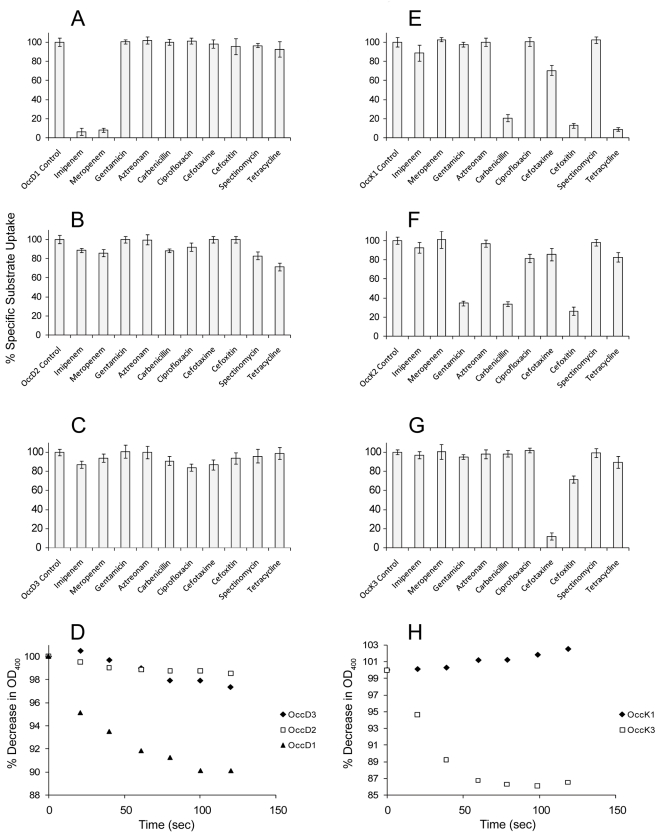
Binding and transport of antibiotics by Occ channels is very specific. Effect on arginine (A–C), benzoate (E), glucuronate (F), and pyroglutamate (G) uptake in the presence of a 10-fold excess of various antibiotics. (A) OccD1, (B) OccD2, (C) OccD3, (E) OccK1, (F) OccK2, and (G) OccK3. In addition, representative liposome swelling assays are shown with imipenem (D) and cefotaxime (H).

A very important but so far ignored point in drug design is the question of how antibiotics traverse the OM of Gram-negative bacteria. This is especially relevant for pseudomonads due to their highly impermeable OM. Since the Occ channels are substrate specific for low-molecular weight compounds (<200 Da), we anticipated that the specificity might be even more pronounced for the higher molecular weight antibiotics (∼300–500 Da; Figure S10). We tested this hypothesis by determining the effect of a number of antibiotics, all of which are effective against *P. aeruginosa* in vivo, on Occ channel-mediated substrate transport. The results shown in Figure 6 for OccD1–3 and OccK1–3 demonstrate that the interactions of the antibiotics with the various channels are indeed highly specific. For OccD1, inhibition of arginine uptake is caused only by imipenem and meropenem, in accordance with the known role of this channel in carbapenem antibiotic uptake [21],[32]–[34]. Thus, remarkably, OccD2 and OccD3, while efficient in arginine uptake (Figure 4), are not uptake channels for carbapenems. In fact, none of the remaining antibiotics interfere substantially with arginine transport by OccD subfamily members (Figures 6A–C and Figure S13). We also tested the uptake of antibiotics directly, using liposome swelling assays. As shown in Figure 6D for imipenem, uptake of this antibiotic is only mediated by OccD1 and not by OccD2/D3, in accordance with the competition data. This is an important result, suggesting that channel-specific screening of antibiotic uptake can be done in vitro in a time-efficient manner by transport competition experiments.

Transport mediated by OccK subfamily members is affected by different antibiotics compared to OccD channels (Figures 6E–G), which is consistent with the different substrate specificities. The carbapenems are generally ineffective, and efficient competition is observed for only a few antibiotics out of the 10 tested. As judged by the inhibition of transport, OccK1 interacts with carbenicillin and cefoxitin. Intriguingly, OccK1-mediated transport is also affected by tetracycline, which is a non-β-lactam polyketide compound that does not have a carboxyl group. OccK2-mediated transport is also affected by carbenicillin and cefoxitin, albeit in a less efficient manner than OccK1. In addition, competition is observed with the amino-glycoside gentamycin, which also does not possess a carboxyl group. Thus, it appears that the strict requirement for a carboxyl group in low-molecular weight substrates may be less stringent in antibiotics, perhaps as a consequence of the many functional groups present in these higher molecular weight compounds (Figure S10). OccK3-mediated transport is affected only with the cephalosporin cefotaxime. OccK3 thus appears to serve as a specific entry channel for this antibiotic, which is borne out by the liposome swelling experiments (Figure 6H). For the other OccK channels, none of the antibiotics affects transport efficiently, with one striking exception: OccK7-mediated transport is lowered very efficiently by meropenem, but not by the closely related imipenem. The observed selectivity of the channels in terms of antibiotic binding and transport is striking. One general implication of these findings is that acquisition of resistance towards many antibiotics may require down-regulation or inactivation of only a few channels, and in some cases perhaps just one. Consequently, antibiotics should ideally be designed to be able to pass through multiple channels.

## Discussion

Our ability to measure efficient diffusion-mediated transport into total membrane vesicles without employing concentrative uptake assays that have been used for ion channels [35] is perhaps surprising, making it important to demonstrate that what we are measuring is in fact transport into and not, for example, substrate binding to the outside of the vesicles. Several arguments can be given. First, substrate-specific channels have generally low affinities for low molecular-weight substrates, often in the (sub) mM range (e.g., ∼2 mM for vanillate binding to OccK1; [23]). In our assays, Occ protein and substrate concentrations are low (sub-µM) and similar to each other, making it unlikely that the signals we observe originate from binding to the channels. A second, important argument that favors transport over binding are the results obtained for the positive controls OmpG and *E. coli* C43 in our transport assays. For most substrates that we investigated, substantial amounts of radiolabel become associated with the vesicles (Figure 4). It is highly unlikely that the signals are caused by binding, since OmpG and OmpF/C (in *E. coli* C43) are non-specific porins that do not bind substrates. Besides these indirect arguments, we have also performed control experiments to show that the accumulation of radiolabel in our assay is due to transport. When the vesicles are treated with polymixin B following substrate uptake, the radiolabel is lost, indicating that polymixin-induced permeabilization results in the loss of substrate from the vesicles (Figure S14). To our knowledge, this is the first time that the efficient transport of various substrates at low concentrations has been reported for outer membrane diffusion channels.

The transport data demonstrate that Occ channels have a pronounced preference for substrates with a carboxyl group, due to the universal presence of a basic ladder on one side of the barrel wall. Beyond the requirement for a carboxyl group, the two Occ subfamilies have very different specificities. OccD channels are highly specific and transport basic amino acids. OccK channels, on the other hand, appear to be less selective and facilitate passage of a wider variety of substances, with a preference for cyclic compounds. Within both subfamilies, a substantial amount of substrate preference overlap is observed.

The ultimate goal of the studies described here is to understand the structural basis of Occ channel specificity on an atomic level. Ideally, such an understanding should allow prediction of substrate/antibiotic preferences, including for channels for which structures are not available. In order to achieve this, co-crystal structures of Occ channels with their substrates are required. Thus far, we have focused on obtaining such structures for OccD1 and OccK1. However, we have not yet been successful for several reasons. First, we have focused on suboptimal substrates since the results of transport experiments were not yet available (i.e., we used vanillate rather than benzoate for co-crystallization with OccK1). Second, as is the case for most diffusion channels, substrate affinities are likely to be relatively low. Therefore, substrate binding under many crystallization conditions such as low pH or high ionic strength may therefore be very weak. Another important reason for the difficulty in obtaining co-crystal structures is our finding that some Occ channels are dynamic and characterized by open and closed states. Consequently, purification and/or crystallization conditions may favor (partially) closed states. Indeed, we crystallized such a closed state for OccD2. In addition, the pores observed for OccD1 and OccD3 are likely too small to allow binding and transport of their substrates. This is especially true for OccD1 and carbapenem antibiotics. In the latter case, even a movement of the insertion in L7 that partially blocks the pore is likely not enough to generate a large enough pore for transport of the antibiotic. Intriguingly, the B-factors for OccD1 suggest that the pore-constricting loop L3 is flexible (Figure S15), and we speculate that movement of this loop could enlarge the pore to allow transport. Thus, at least some Occ proteins may require conformational changes in the loops that constrict the pore to allow substrate binding and passage. Interestingly, it has been reported that the addition of lipopolysaccharide (LPS) to OccD1 in single-channel experiments increased the conductance of this channel ∼20-fold [36], suggesting that addition of LPS to OccD channel preparations may stabilize an open state. In any case, the availability of many Occ channels in milligram quantities, together with the identification of better substrates reported here, will facilitate the generation of co-crystal structures.

**Figure 7:**
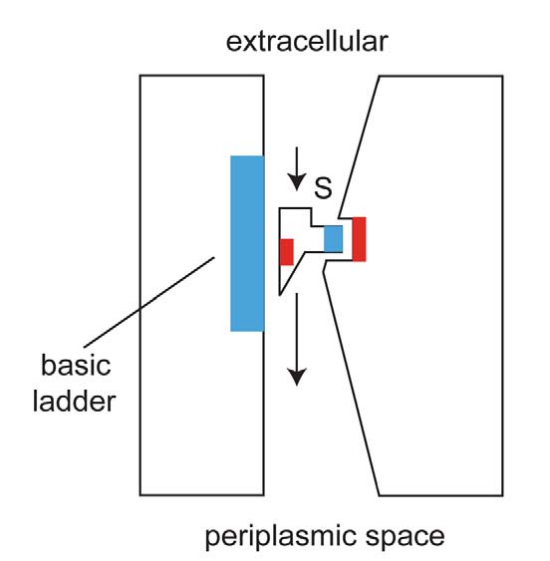
Schematic model of transport mediated by OccD1. Substrate selection occurs by matching of opposite charges (blue, positive; red, negative) present on the substrate and in the constriction of the channel.

In the absence of a channel-substrate co-crystal structure, some general rationalizations regarding substrate recognition can still be made. For example, OccD1 has a distinct, asymmetric charge distribution across the pore constriction reminiscent to that observed in the *E. coli* porins OmpF and OmpC. The side of the basic ladder forms a positively charged surface, whereas the opposite side of the channel features a negatively charged pocket due to the presence of a carboxyl group, hydroxyl groups, and a number of backbone carbonyls [23]. Thus, the observed substrate requirement of OccD1 for a carboxyl group and a positively charged group some distance away can be explained in a qualitative way by the structure (Figure 7), even if the structure does not resemble the fully open channel. Interestingly, recent molecular dynamics simulations suggest that the transient interaction of ampicillin with OmpF is similar to what we propose for OccD1 and imipenem/meropenem, with interacting opposite charges on the substrate and in the constriction of the channel (Figure 7) [37].

Our data explain why soil bacteria such as pseudomonads are adept at growth under nutrient-poor conditions. The uptake experiments, performed with very low substrate concentrations, suggest that the Occ channel family as a whole can mediate the efficient uptake of a substantial number of low molecular weight compounds. Since each family member transports only a limited number of substances, the OM remains an effective permeability barrier as evidenced by the generally low effectiveness of antibiotics permeation through the Occ channels. Our results also help to rationalize the adaptations of bacteria in response to antibiotics treatment and the emergence of acquired resistance. A well-studied example of this is the response of *P. aeruginosa* to carbapenem antibiotics such as imipenem and meropenem. Clinical isolates of *P. aeruginosa* resistant to these compounds often show, in addition to upregulation of efflux pumps and/or expression of β-lactamases, the disappearance of OccD1 [32],[33], which is normally one of the more abundant OM proteins. Although the physiological substrates of OccD1 are unknown, the overlapping substrate specificities with other OccD subfamily members (at least with respect to arginine; Figure 4) suggest that a lack of OccD1 in the carbapenem-resistant *P. aeruginosa* strains is unlikely to affect fitness greatly.

Until now, the screening of antibiotics in drug design has largely focused on the interaction of the compound with their intracellular targets. However, the effectiveness of antibiotics in Gram-negative bacteria also depends critically on the balance of three fundamental processes that occur prior to target interaction: influx mediated by OM channels, efflux mediated by efflux pumps, and degradation mediated by periplasmic and cytoplasmic enzymes. It is logical to assume that efficient antibiotics will have enhanced permeation properties, and the rational design of such compounds will require knowledge of how they interact at the atomic level with the OM channels that mediate their passage. Surprisingly, it is completely unclear how antibiotics interact with and pass through OM channels, with the exception of *E. coli* OmpF [36]. Moreover, for virtually all antibiotics it is also unknown which channel(s) are utilized for OM permeation in vivo. Our characterization of Occ channel structure and specificity is a first step towards understanding the interaction of antibiotics with OM channels of *P. aeruginosa*. The use of an integrated approach encompassing genomics, biochemistry, and structural/computational biology may allow the rational design of novel antibiotics in the future, which will greatly help the fight against emerging bacterial resistance.

## Materials and Methods

### Cloning, Expression, and Purification of Channels

The *occ* genes from *P. aeruginosa* and *fadL* and *ompG* genes from *E. coli* were cloned into the *E. coli* expression vector pB22 [38],[39] with an N-terminal hexa-histidine tag for purification. DNA sequencing was performed at CFAR DNA sequencing facility (UMass Medical School, Worcester, MA). BL21(DE3) T1 phage-resistant cells (New England Biolabs, Ipswich, MA) were transformed with pB22-Occ or pB22-OmpG/FadL constructs. The cells were grown to OD_600_∼0.6 at 37°C and then induced with 0.1% arabinose at 20°C overnight. Cells were harvested by centrifugation at 4,500 rpm for 30 min (Beckman Coulter, J6-MC). Cell pellets were suspended in TSB (20 mM Tris, 300 mM NaCl, 10% glycerol, pH 8.0) and cells were lysed by sonication (3×40 s intervals) (Branson Digital Sonifier). Total membranes were obtained by centrifugation at 40,000 rpm for 40 min (45 Ti rotor, Beckman L8-70M ultracentrifuge). Membranes were homogenized in TSB and solubilized in 1% DM (n-Decyl-β-D-Maltopyranoside, Anatrace, Santa Clara, CA) and 1% LDAO (n-Dodecyl-N,N-Dimethylamine-N-Oxide, Anatrace) for 2 h at 4°C followed by centrifugation at 40,000 rpm for 30 min to remove precipitates and unsolubilized membranes. The membrane extract was applied to a 10 ml nickel column. The column was washed with 10 column volumes (CV) TSB containing 0.2% DM and 15 mM imidazole. The proteins were eluted with 3 CV TSB containing 0.2% DM and 200 mM imidazole. The proteins were further purified by gel filtration chromatography using 10 mM Tris, 50 mM NaCl, and 0.12% DM, pH 8.0. For final polishing and detergent exchange, another gel filtration chromatography step was performed. The buffer used for this column varied depending on the protein; for most of the channels, 10 mM Tris, 50 mM NaCl, and 0.3%–0.35% C_8_E_4_ was used at pH 8.0. Typically, small amounts (0.02%–0.1%) of another detergent (either LDAO, β-OG, DM or diheptanoyl-phosphatidylcholine) were added to modify channel solubility and crystallization properties. The purified proteins were concentrated to 5–15 mg/ml using 50 kDa molecular weight cutoff filters (Amicon) and directly flash-frozen in liquid nitrogen, without dialysis.

### Crystallization of Occ Channels and Structure Determination

Initial crystallization trials were performed at 295 K using a Gryphon crystallization robot (Art Robbins Instruments) and by sitting-drop vapor diffusion, using various commercial and in-house screens. If required, the initial hits were optimized by fine-screening with larger drops using various protein-to-mother liquor ratios by hanging drop vapor diffusion. Depending on the conditions, crystals were flash-frozen in liquid nitrogen either directly from the drop or after suitable cryoprotection, typically by adding glycerol to the drop. The crystals that gave suitable diffraction data were obtained under the following conditions: OccD1: 2 M (NH_4_)_2_SO_4_, 50 mM Na-citrate pH 5.5; OccD2: 20% PEG 3350, 15 mM Tricine pH 8.5; OccD3: 25% PEG 1 K, 0.2 M (NH_4_)_2_SO_4_, 50 mM Na-citrate pH 4.0; OccK1: 25% PEG 400, 0.1 M Na-citrate, 50 mM NaCl, 25 mM vanillate pH 6.2; OccK2, 25% PEG 1 K, 50 mM Li_2_SO_4_, 50 mM Na_2_SO_4_, 50 mM Na-acetate pH 5.5; OccK3: 30% PEG 1 K, 0.2 M 0.2 M (NH_4_)_2_SO_4_, 0.1 M Hepes pH 7.0; OccK4: 30% PEG 400, 50 mM NaCl, 0.1 M Na-citrate pH 5.5; OccK5: 35% PEG 400, 0.1 M MgCl_2_, 50 mM Tris pH 8.5; OccK6: 33% PEG 1 K, 0.2 M (NH_4_)_2_SO_4_, 50 mM Na-acetate pH 5.5.

Diffraction data were collected at 100 K at the National Synchrotron Light Source (Brookhaven National Lab) at beamlines X6A and X25. Processing was carried out with HKL2000. The Occ channel structures were solved by molecular replacement in Phaser [40] using either OccD1 (PDB ID: 2ODJ) or OccK1 (PDB ID: 2QTK) as the search models, depending on the sequence identity with the channel under study. Model (re)building was performed manually within COOT [41], and the structures were refined using Phenix [42]. Structure validation was performed within Phenix.

### Single-Channel Current Recordings on Planar Lipid Bilayers

Single-channel current measurements were carried out with planar lipid membranes [43],[44]. Briefly, both chambers (1.5 ml each) of the bilayer apparatus were separated by a 25-µm-thick teflon septum (Goodfellow Corporation, Malvern, PA). An aperture in the septum of ∼60 µm diameter was pretreated with hexadecane (Aldrich Chemical Co., Milwaukee, WI), which was dissolved in highly purified n-pentane (Burdick & Jackson, Allied Signal Inc., Muskegon, MI) at a concentration of 10% (v/v). The lipid bilayer was formed with 1,2-diphytanoyl-*sn*-glycerophosphocholine (Avanti Polar Lipids Inc., Alabaster, AL, USA). The standard electrolyte in both chambers was 1 M KCl, 10 mM potassium phosphate, pH 7.4. The proteins were added to the *cis* chamber, which was at ground. Current flow is shown as positive and it represents a positive charge moving from the *trans* to *cis* chamber. Currents were recorded by using an Axopatch 200B patch-clamp amplifier (Axon Instruments, Foster City, CA) connected to the chambers by Ag/AgCl electrodes. An Optiplex Desktop Computer (Dell, Austin, TX) equipped with a Digitdata 1440 A/D converter (Axon) was used for data acquisition. The output from this amplifier was also filtered by an 8-pole low-pass Bessel filter (Model 900, Frequency Devices, Haverhill, MA) at a frequency of 10 kHz and sampled at 50 kHz. Data acquisition and analysis was performed using pClamp 10.2 software (Axon).

### Preparation of *E. coli* Membrane Vesicles

BL21 omp8 (Δ*lamB ompF*::Tn*5* Δ*ompA* Δ*ompC*) cells [45] were transformed with pB22-Occ or pB22-OmpG/FadL constructs. 1 L of cells were grown to OD_600_∼0.6 at 37°C in LB media and then induced with 0.1% arabinose at 20°C overnight. Total membrane vesicle preparations were carried as described previously [46] with some modifications: *E. coli* cells were collected by centrifugation at 4,200 rpm for 12 min (Beckman Coulter, J6-MC). Cells were re-suspended in 40 ml of 10 mM HEPES pH 7.0. In order to protect outer membrane lipid integrity, EDTA/Lysozyme lysis was replaced with brief sonication for 40 s (Branson Digital Sonifier). Unbroken cells were removed by centrifugation at 9,000 rpm for 15 min (45 Ti rotor, Beckman L8-70M ultracentrifuge). The cleared suspension was centrifuged at 40,000 rpm for 40 min (45 Ti rotor, Beckman L8-70M ultracentrifuge), and the supernatant containing the cytoplasmic and periplasmic proteins was removed. The *E. coli* total membranes were resuspended in 1 ml of 10 mM HEPES pH 7.0 Membrane vesicles were created by sonication [47] in a bath sonicator (Model G112SPIT, Laboratory Supplies Co., Hicksville, NY) for 15 min to facilitate vesicle formation. In order to obtain a more homogeneous vesicle population, the vesicles were extruded (Avanti mini extruder, Avanti Polar Lipids, Alabaster, AL) using 0.4 micron nucleopore track-etch membrane filters (Whatman). The vesicles were divided into 100 µl aliquots, flash frozen in liquid N_2_ and stored at −80°C. Since multiple freeze-thaw cycles have a negative effect on transport (Figure S7), we always used fresh aliquots of vesicles, which were thawed on ice prior to the assays.

### Quantification of Outer Membrane Channels in Vesicles

The total protein concentrations in membrane vesicles were determined using the BCA Protein Assay Kit (Thermo Scientific, Rockford, IL). OmpG, FadL, and Occ channel quantities in membrane vesicles were determined by comparison of the band intensities in Western blots with that of purified OccD1, using the Kodak 1D 3.6 digital imaging program (Eastman Kodak Company, Rochester, NY). For quantification of the proteins in the Western blots, the histidine tag was detected using Penta-His HRP conjugate (Qiagen, Germantown, MD). For the C43(DE3) strain, the combined expression levels of the untagged endogenous porins OmpC and OmpF were compared with that of OccD1 using sarkosyl-extracted outer membrane preparations in Coomassie-stained SDS-PAGE. The protein quantitations were used to normalize the uptake levels in our transport assays, thus yielding specific transport activities.

### Radiolabeled Substrate Uptake Assays

Radiolabeled substrates ^14^C-arginine was purchased from PerkinElmer (Boston, MA); ^14^C-phenyacetic acid, ^3^H-pyroglutamic acid, and ^14^C-citric acid from Moravek Biochemicals (Brea, CA); and ^3^H-glucuronic acid, ^3^H-glucose, ^3^H-benzoate, and ^14^C-vanillate from American Radiolabeled Chemicals (St Louis, MO). Total membrane vesicles were diluted to a 1 mg/ml total membrane protein concentration in 10 mM HEPES, pH 7.0 buffer. Using these conditions, the concentrations of the various Occ proteins in the assay varied between 0.2 and 0.7 µM. Radiolabeled substrates were diluted to 25 µM (^14^C-arginine) or 50 µM (^3^H-benzoate, ^3^H-glucuronic acid, ^3^H-glucose; ^3^H-pyroglutamic acid) in 10 mM HEPES, pH 7.0 buffer. Uptake experiments were started with the addition of the 1 µl radiolabeled substrate to 100 µl of membrane vesicles. The vesicles were briefly vortexed and centrifuged at 3,000 rpm for 5 s (Centrifuge 5415D, Eppendorf, Hauppauge, NY) to collect the contents at the bottom of the microcentrifuge tube. The incubation was carried out at 25°C for the times mentioned in the figure legends. Following incubation, the membrane vesicles were filtered using 0.22 micron nitrocellulose filters (Millipore, Billerica, MA) on a vacuum filtering apparatus (Model FH22V, Hoefer Inc., San Francisco, CA), and washed 3 times with 1 ml of 10 mM HEPES, PH 7.0 buffer. The filters were then placed in scintillation vials containing 5 ml Econo-Safe scintillation fluid (Atlantic Nuclear Corp., Rockland, MA) and counted using a LS 6500 multi-purpose scintillation counter (Beckman Coulter, Brea, CA). Filter backgrounds were determined as follows: 100 µl of 0.25–0.5 µl radiolabeled substrate in 10 mM HEPES, pH 7.0 was filtered using 0.22 micron nitrocellulose filters on a vacuum filtering apparatus and washed 3 times with 1 ml of 10 mM HEPES, PH 7.0 buffer. The filters were then placed in scintillation vials containing 5 ml Econo-Safe scintillation fluid (and counted using a LS 6500 multi-purpose scintillation counter). Filter backgrounds were subtracted from signals obtained for samples.

### Radiolabeled Substrate Uptake in the Presence of Unlabeled Potential Substrates and Antibiotics

Total membrane vesicles were diluted to a 1 mg/ml total membrane protein concentration in 10 mM HEPES, pH 7.0 buffer. For OccD1, uptake was started by addition of 1 µl of 250 µM or 2.5 mM (Table S3) of unlabeled substrate or antibiotic, and 1 µl of 25 µM ^14^C-Arginine into 100 µl of vesicles, and incubation was carried out for 15 min at 25°C. For OccK1 and OccK2, the uptake assay was started by addition of 1 µl of 500 µM or 5 mM (Table S3) of unlabeled substrate (or antibiotic) and 1 µl of 50 µM of either ^3^H-benzoate (OccK1) or ^3^H-glucuronic acid (OccK2) into 100 µl of vesicles, and incubation was carried out for 10 min at 25°C. Filtering, washing, and counting of radioactive signal in each uptake assay was carried out as described above.

### Vesicle Leakage Assays

*E. coli* membrane vesicles were checked for leakage by calcein, a water-soluble, self-quenching fluorescent dye [48]. Vesicles entrapped with calcein were prepared as follows: *E. coli* membranes were obtained as described above and the membranes were resuspended in 1 ml of 10 mM HEPES, 70 mM calcein, pH 7.0, and sonicated in bath sonicator for 15 min. After extrusion using 0.4 micron nucleopore track-etch membrane filters, the vesicles were flash-frozen in liquid N_2_ once and thawed on ice. Non-entrapped calcein was separated from vesicles on a mini Sephadex G25 column. The fluorescence of 100 µl of vesicles was followed for 10 min using a Tecan Safire plate reader (Mannedorf, Switzerland) with excitation and emission filters at 490 and 520 nm, respectively.

### Liposome Swelling Assays

Osmotically active liposomes were prepared as described previously [20] with slight modifications. Liposomes were made by suspending a dried film containing 6 µmol egg phosphatidylcholine (Avanti Polar Lipids, Alabaster, Al), 0.3 µmol of diacetylphosphate (Sigma, St. Louis, MO), and 10 µM Occ protein in 10 mM HEPES, pH 7.0 containing 12 mM stachyose (Sigma). The suspension was then sonicated in bath sonicator (Model G112SPIT, Laboratory Supplies Company, Hicksville, NY) until translucent. For the uptake assays, 2 µl of the proteoliposomes were added to 100 µl of antibiotic solutions in 10 mM HEPES, pH 7.0. Liposome swelling was measured in triplicate by monitoring changes in optical density at 400 nm at 17 s intervals for 600 s. Readings were normalized to the maximal optical density for each time course. The antibiotic concentration isosmotic to the intraliposomal milieu was determined by identifying the concentration of antibiotic that did not elicit liposomal swelling upon dilution in control liposomes (liposomes without protein). The isosmotic concentration of antibiotics ranged from 10 mM to 15 mM. Transport of each antibiotic through proteoliposomes was assayed at the predetermined isosmotic concentration.

### Accession Codes

The atomic coordinates and structure factors for the Occ channels have been deposited in the Protein Data Bank with accession numbers 3SY7 (OccD1), 3SY9 (OccD2), 3SYB (OccD3), 3SYS (OccK1), 3SZD (OccK2), 3SZV (OccK3), 3T0S (OccK4), 3T20 (OccK5), and 3T24 (OccK6).

## Supporting Information

Figure S1 CLUSTALW multiple sequence alignment of OccD/OccK channels. Invariant residues (*), highly conserved residues (:), and conserved residues (.) are shown at the bottom. The observed secondary structure elements for OccD1 and OccK1 are shown (β strands colored as blue arrows for OccD1 and as salmon arrows for OccK1; α-helices in red). The missing residues in the crystal structures are shown as hatched orange bars. The residues that line the pore constriction are highlighted in green. Amino acid residues that are part of the basic ladder are highlighted in pink. Pore lining basic residues that are also part of the basic ladder are highlighted in purple. The L7 insertion for OccD1 is highlighted in yellow and the N-terminal extension of OccD3 is shown in cyan.
(PDF)

Figure S2 Variable pore openings for OccD channels. (A) The small OccD3 pore as present in the crystal structure and (B) the large pore that could potentially be formed after movement of the N-terminal extension (shown in red; residues 1–30), for example as a result of high ionic strength in the single channel conductance experiments. (C) The small pore in the current structure of OccD1. Note the different conformation of the insertion in loop L7 (colored green) compared to that in the original OccD1 structure (PDB ID: 2ODJ). As a consequence, OccD1 in the original structure has a larger pore (D). In all cases, the view is from the extracellular side.
(PDF)

Figure S3 Occ channels have differently sized shape and pores. (A) Wire diagrams from the side made with HOLE [1], showing the surface of the Occ channel pores in blue. Areas of the pores with a diameter smaller than 4.5 Å are colored green. The extracellular side is at the top. The pores for OccD1 and OccD2 could not be calculated. (B) Pore radii plotted as a function of the pore coordinate z. Channels were aligned to OccD3 and radii calculated using HOLE. Due to the complex pore shape and absence of a pore for OccD1 and OccD2, respectively, profiles for these two channels could not be calculated.
(PDF)

Figure S4 Single-channel electrical recordings of (A) OccD4, (B) OccD5, (C) OccD6, and (D) OccK7. The data were collected at an applied transmembrane potential of +80 mV. The buffer solution in the chamber contained 1 M KCl, 10 mM potassium phosphate, pH = 7.4. For the sake of clarity, the single-channel electrical traces were low-pass Bessel filtered at 2 kHz. The numbers located above the traces represent the single-channel conductance of the most probable sub-state of the channel. The averages were derived from at least three independent single-channel electrical recordings.
(PDF)

Figure S5 Comparison of ^3^H-arginine uptake in OccD1 proteoliposomes made using either *E. coli* or *P. putida* lipids. 0.25 µM radiolabeled arginine was added to proteoliposomes and the transport reaction was stopped after 15 min. ^3^H-arginine uptake in *P. putida* proteoliposomes containing internal *E. coli* LAO-binding protein (Lysine-Arginine-Ornithine binding protein) is also shown. LAO-binding protein inside the proteoliposomes potentially forms a “sink” for arginine transport.
(PDF)

Figure S6 Expression levels of channels in total membrane vesicles. Representative Western blots of vesicles containing Occ channels, FadL, and OmpG: (1) OccD4; (2) OccK1; (3) OccD2; (4) OccK2; (5) OccD1; (6) Pure OccD1 (Control); (7) OccK5; (8) OccD5; (9) OccK4; (10) OccK3; (11) OccD3; (12) OccD6; (13) OccK7; (14) OccK6; (15) Pure OccD1 (Control); (16) OmpG; and (17) FadL. Vesicles were diluted to contain 2 µg total membrane protein and the band intensity from Occ channels was compared to that of 0.1 µg purified OccD1 (control).
(PDF)

Figure S7 Right-side-out orientation of Occ channels in membrane vesicles. (A) Effect of freeze-thaw cycles on specific substrate transport activity (0.25 µM ^3^H-arginine, 15-min assay by OccD1 vesicles). The specific activity decreases upon freeze-thawing, presumably as a result of random reorientation of OccD1 channels in the vesicles. (B) Detection of the OccD1 N-terminal histidine tag by anti-His antibody. The tag, originally inaccessible to the antibody (i.e., lumenal), becomes more accessible upon freezing-thawing.
(PDF)

Figure S8 Optimization of substrate uptake by Occ channels in total membrane vesicles relative to “empty” background (pB22). Assays contain varying concentrations of radiolabeled substrates (0.05–10 µM) as indicated in the figure. Uptake times were as follows: arginine, 15 min; benzoate, 10 min; glucuronate, 10 min; pyroglutamate, 15 min; vanillate, 40 min; glucose, 15 min; citrate, 40 min; and phenylacetate, 40 min.
(PDF)

Figure S9 Time curves of arginine uptake by OccD1 at varying concentrations of substrate. Arginine was added to the uptake assays at the final concentrations indicated in the figure and samples were taken at 2, 5, 10, 20, and 30 min.
(PDF)

Figure S10 Structures of (A) radiolabeled Occ channel substrates and (B) antibiotics used for uptake competition experiments in this study. Compounds that do not contain a carboxyl group are labeled in red.
(PDF)

Figure S11 Uptake of non-preferred radiolabeled substrates in *E. coli* Bl21 omp8 total membrane vesicles, expressing empty plasmid (pB22), Occ channels, or *E. coli* OmpG or FadL. Substrates are (A) vanillate (10 µM, 45 min), (B) phenylacetate (10 µM, 45 min), and (C) citrate (10 µM, 45 min). 100% specific activities correspond to 27.8±1.7 (A), 7.6±0.5 (B), and, 6.2±0.6 (C) pmoles substrate/min/mg protein.
(PDF)

Figure S12 Vanillate binding sites in the OccK1 structure. (A) Cartoon overview from the side, showing the two vanillate molecules as stick models (carbons, yellow; oxygens, red). For orientation, the central basic ladder residue Arg381 lining the pore constriction is also shown. The extracellular side is at the top of the figure. (B) Stereo diagram showing the binding pocket for the periplasmic vanillate molecule (VAN 2). Electron density (2F_o_–F_c_ map, contoured at 1.5 σ) is shown as a blue mesh.
(PDF)

Figure S13 Antibiotic transport by Occ channels. Arginine (A–B) and benzoate (C–F) uptake measured in the presence of a 10-fold excess of antibiotics. The following channels are shown: OccD5 (A), OccD6 (B), OccK4 (C), OccK5 (D), OccK6 (E), and OccK7 (F).
(PDF)

Figure S14 Effect of vesicle permeabilization on arginine transport. (A) Arginine uptake by OccD1 vesicles in the presence of varying concentrations of polymyxin B. 100% corresponds to arginine uptake measured with OccD1 control vesicles that were not incubated with polymyxin B. (B) Amount of vesicles remaining on the filter as measured by total protein quantification of OccD1 control vesicles (100%) and OccD1 vesicles incubated with 0.5% polymyxin B.
(PDF)

Figure S15 Backbone cartoon views of OccD1 and OccK1 viewed from the extracellular side colored by B-factor (blue, low B-factors; red, high B-factors). Note the high B-factors for the L3 loop and L7 insertion (L7i) for OccD1 relative to the barrel wall, indicating possible mobility of these segments.
(PDF)

Table S1 Proposed nomenclature change for Occ channels.
(PDF)

Table S2 Data collection and refinement statistics of Occ channels.
(PDF)

Table S3 Definition of Occ channel specificity. Uptake of radiolabeled arginine by OccD1, benzoate by OccK1, and glucuronate by OccK2 was measured in the presence of a 100-fold excess of unlabeled low-molecular weight compound. In all cases, total levels of uptake are reported, expressed as a percentage of uptake in the absence of unlabeled compound (100%). Substrates containing a carboxyl group are italicized.
(PDF)

Text S1 Presentation and discussion of the revised nomenclature for Occ proteins and detailed descriptions of methods and a supporting reference.
(PDF)

## Abbreviations

CV: column volumes
LPS: lipopolysaccharide
Occ: Outer membrane carboxylic acid channel
OM: outer membrane
