# Supplementary material for: Substrate Specificity within a Family of Outer Membrane Carboxylate Channels
Source: PLoS Biol. 2012 Jan 17;10(1):e1001242. doi: 10.1371/journal.pbio.1001242 (PMC3260308; doi:10.1371/journal.pbio.1001242)
Supplement: Figure S1 — CLUSTALW multiple sequence alignment of OccD/OccK channels. Invariant residues (*), highly conserved residues (:), and conserved residues (.) are shown at the bottom. The observed secondary structure elements for OccD1 and OccK1 are shown (β strands colored as blue arrows for OccD1 and as salmon arrows for OccK1; α-helices in red). The missing residues in the crystal structures are shown as hatched orange bars. The residues that line the pore constriction are highlighted in green. Amino acid residues that are part of the basic ladder are highlighted in pink. Pore lining basic residues that are also part of the basic ladder are highlighted in purple. The L7 insertion for OccD1 is highlighted in yellow and the N-terminal extension of OccD3 is shown in cyan. (PDF) [file pbio.1001242.s001.pdf]

```
OccD1     -------------------------DAFVSDQAEAKGFIEDSSLDLLLRNYYFNRDGKSG 35
OccD2     -----------------------------DEGEAKEGFIEGSSLQLLTRNYYFNHDRRHA 31
OccD3     ADEQENPPAPDNPSYAAEVQSIPSVAKPIKGQAGATGLVEGQSLTLTTRNFYSRENMKDS 60
OccD4     ---------------------------EENAERSKEGFIEGSEVNLLLRNFYFNRDFRKG 33
OccD5     ---------------------------------GDGGFVEDSELQFLARTYYFNRDYRDS 27
OccD6     -----------------------------NDQEAAKGFVEDSHLDLFFRNGYISRDYKHG 31
OccD7     -------------------------------ATEEAKAPDYLEWRLLNRNYFLYNDYRR- 28
OccD8     ----------DDSLGHRDNISTGLNQRQKAEMKVPPGFVEGSTLNGFIRNYYFARDNHDT 50

OccK1     ---------------------------------AEGGFLEDAKTDLVLRNYYFNRDFRDH 27
OccK2     -----------------------------GHVHAGQGFLEDAKASLTARNFHLHRNFVG- 30
OccK3     ------------------------------------DLVEDSHASLELRNFYFNRDFRQS 24
OccK4     ------------------------------------EFLADSSAHLDLRNFYQLRDYRQH 24
OccK5     -----------------------------------AGFLEDSKASLETRNFYMNRDFRD- 24
OccK6     ------------------------------------EFIKDSKASIELRNFYFNRDFRQE 24
OccK7     ------------------------------------EFFADGKAGLELRNFYFNRDYRQP 24
OccK8     -----------------------------------AGFIEDSKASLTLRNFYINTDNRNG 25
OccK9     -----------------------------------EGFIEDASVSLGLRNLYFNRDFRQP 25
OccK10    ------------------------------------AFLEDGSARLEARTVYFNRDFRDG 24
OccK11    -----------------------------------EGFLEDSRASLALRNFYMNRDFRD- 24
                                                      *.  .    :

OccD1     ----SGDR-----------VDWTQGFLTTYESGFTQGTVGFGVDAFGYLGLKLDGT--SD 78
OccD2     SG-HDS-------------KEWAQGFIATFQSGYTPGVVGFGVDAYGMLGLKLDGG--GG 75
OccD3     FTFRIPKAGG-GSQRIHQRNAWVQGTVLKYSSGYTQGTVGFGFDVAAFNEIALERG--KG 117
OccD4     QSS-PAGGGY--------TEEWVQGFMANFSSGFTQGTLGVGIDAFAQLGVRLDSG--GG 82
OccD5     PNNAGRNRFKPRSERNGYREEATQGLRLQFASGYTPGSLGFGLDAHAMLGLQLDSG--GG 85
OccD6     ----RQDK-----------AEWGQAATATFTSGFTQGTVGVGVDAFGLYAVRLDGG--KG 74
OccD7     -ADAGR----------GYRKEWAHGLIVDLESAYTPGRVGFGLDLHGFAGFKLDGG--RG 75
OccD8     PSRRDQ-------------REWAQGLMLSFRSGYTDTPIGIGLDAHAFYGLRLDGG--GG 95

OccK1     ----DAGKSL--------VDEWAQGFILKFSSGYTPGTVGVGLDAIGLFGVKLNSG--RG 73
OccK2     ----DASQGK--------AEEWTQSFILDARSGFTQGSVGFGLDVLGLYSLKLDGG--KG 76
OccK3     ----GARDN---------ADEWAQGFLLRLESGFSEGTVGFGVDAIGLLGFKLDSG--SG 69
OccK4     ----DAPQSQ--------AGNWSQGFVLRLQSGFTGGPLGFGLDATGLLGVKLDSG--RG 70
OccK5     ----GPGQSK--------REEWAQGFILNLQSGYTQGTVGFGLDAMGMLGVKLDSG--RG 70
OccK6     ----GASQSK--------AEEWAQGFLLRYESGYTEGTIGFGVDAIGLLGVKLDSS--PD 70
OccK7     ----GASQSY--------SEEWAQGFLLRYESGYTEGLFGLGVDALGLLGVRLDSS--PE 70
OccK8     ----TASPSK--------QEEWGQGFILNYQSGFTQGTVGFGVDALGLLGVRLDGGGRAG 73
OccK9     ----GAAQSK--------QEEWAQGFLLQAKSGYTQGTLGLGVELIGQLGLKLDSS--PD 71
OccK10    SSANPQGASK--------REEAAQGFILDLRSGYTEGALGFGVDTLAMLGIKLDSS--PA 74
OccK11    ----GAGRAK--------SEEWAQGFLFDYRSGYTEGTLGVGLDLLGKLGVRLDSG--AG 70
                                :.       *.::   .*.*.:   .    . *:

OccD1     KTGT----GNLPVMN----DGKP---RDDYSRAGGA-VKVRISKTMLKWGEMQPTAPVFA 126
OccD2     TGGT----SILPITSPSKEGYESGKAPDEFSSGGAA-LKIRAFDTELKLGDQFLSNPVVA 130
OccD3     RIGGG---GNRTLAN------SDGEALGEWSKLGVANIRLRASNTEFKAGRFLVNTPVFS 168
OccD4     RSGAGGSVDLLPYDD-------QGRPQDDYSRAGGA-VKLRWYGTVLRVGDVFPETPVIQ 134
OccD5     RTGT----GNLPVGA-------DGHPDHRYGKVGGA-LRLRHGETRLKYGQTTTSAPVFA 133
OccD6     RSGA----AGIDFFK----QGDSGSAADDLSKGGAA-VKFRISNTVLKYGDQMPSLPVLS 125
OccD7     HAGT----GLLPLDS-------SGDNASEYSDAGAA-LKLRLGESLLKVGEMTVETPVFD 123
OccD8     SGGA----GVLPLDS-------AGRPADSFSAAGAA-LKLRGLDSLLKIGDQLLENPVIA 143

OccK1     TSNS----ELLPLHD-------DGRAADNYGRVGVA-AKLRVSASELKIGEMLPDIPLLR 121
OccK2     TAGT----QLLPIHD-------DGRPADDFGRLAVA-GKLRVSNSELKIGEWMPVLPILR 124
OccK3     SGGT----GLLPADG------SAGGSQDDYAKLGLT-AKARVSNSLLKVGALHFKSPLVS 118
OccK4     RSND----GTLPFGA------NSKEPVDDYSHLGLT-AKLRYSQTQLQVGILMPQLPVAF 119
OccK5     RSGT----GLLPKD-------SDGRAPDTYSKLGLT-AKVKVSQSELKVGTLIPKLPSVQ 118
```

```
OccK6           RSGT----GLLKRDR------ETGRAQDDYGEAGIT-AKLRASKSTLKIGTLTPKLPVIM 119
OccK7           RSGS----GLLPYST------SDRRAAHDYSSLGLT-AKLRVSHSTLKIGTLMPRLPVVQ 119
OccK8           KSGLDRQPGTVFPLE------SNGEPVHDFASLGLT-AKAKVSNTEFRYGTLQPKLPVVT 126
OccK9           RAGS----GLLPRHA-------DGRAADDYARLGVA-PKLKLSNTELKLGELLPELPILL 119
OccK10          DSNS----GLLPSSGH-----DPRRSVDQYAKAGVA-GKMRFSQTQFRYGAMLPDMPLLK 124
OccK11          RSGT----GLLPLR-------DDGSAAGDYARLDAT-AKLRLSRSELKVGGLVPKLPTIQ 118
                  .                           .    :  : :   : ::  *      *

OccD1           AGGSRLFPQTATGFQLQSSEFE-GLDLEAGHFTEGKEPTTVKSRGELYATYAGET----- 180
OccD2           GGESRMLPQTFRGVSLTNNSFE-DLTLTAG-QVSFTKYYNQSGHRRLGSYYG-----ELP 183
OccD3           YIDNRALPSSFTGFAVTSEELD-NLSLQAGSFRKVSPRTGS-GDEDMTTEYGTRQVK--- 223
OccD4           YGNSRLFPSSFRGFTLVNDSLAKGLTLQAGKLNSMTQPNSTSGSDDFYSFYTGRRID--- 191
OccD5           ASSNRTLAGMAYGLLLEDRSFD-GLLLEGGRFTAASGPGESKVRGDISTVYG----RLGA 188
OccD6           YDNSRLLPESYSGTLITSKEIE-GLELNAGRFT-------AESRKSAEGRDSGG------ 171
OccD7           TSDKRLQPQYARGFFLQSDDLD-RVRIQAGRFTAFKEQASSSGHGDFDG-YG----ASTA 177
OccD8           SGVSRMVPQSYRGVTLKNYHFR-ALELDAG-FVEATRLRNQSGHSHLTSGYGNGTKGGIA 201

OccK1           YDDGRLLPQTFRGFAVVSRELP-GLALQAGRFDAVSLRNSA-DMQDLSAWSAPTQK---- 175
OccK2           SDDGRSLPQTFRGGQLSANEIA-GLTLYAGQFRGNSPRNDA-SMQDMSLFGRPAAT---- 178
OccK3           ANDTRLLPELFRGALLDVQEID-GLTLRGAHLDRNKLNSSS-DYQVFSANRIGGR----- 171
OccK4           RDDVRLLPQTFDGALLTSSEIE-GLTLTAGQLWKSRTRESA-GSDDMYIMGRDKAH---- 173
OccK5           PNNGRIFPQIFEGALLTSKEIK-DLGFTAGRLEKTKIRDSS-DSEDLALNDKNGRFAGVS 176
OccK6           PNDSRLLPQTFQGGALNSMEID-GLTLDAGRLKKVNQRDSS-DNEDMTITGGGKRNIVVR 177
OccK7           FNDTRLHPQTFQGGLLEVNEID-GLALQFGQLRQVKQRDST-NAEDLGITRGNKRNVLAG 177
OccK8           YNDGRLLPVTFEGGQVTSTDLK-DFTLVAGQLEHSKGRNST-DNRSLSIAGANGSSASSR 184
OccK9           RNDGRLLPQTFQGGMLTSREIA-GLTLHGGQMRSLSQRNSS-DHQDLSVDGRGGAF---- 173
OccK10          YNDGRLLPTLFHGAQLTSEEIA-GLRFSATRLERYTARDSS-DAQDIRLHCKNKRYACDT 182
OccK11          PNYGRLFPQVFQGALLTSGELS-GLSLNLGRLTEVSQRNEA-GTSDLALFNRNRRFAGAA 176
                    *  .    *  :    :   . :

OccD1           ----AKSADFIGGRYAITDNLS-ASLYGAELEDIYRQYYLNSNYTIP-LASDQSLGFDFN 234
OccD2           GDRDSHHLSWLGGTWGGIEGFT-SSLYAAELQNVWKQYYADVDYTYE-IDDNWSLNPGAH 241
OccD3           ----GDRLNYLGGNYKPLDGLE-ISLYGSHFQDVWNQYYLGVTHDIGDLENGIALRTAFN 278
OccD4           ----SPWVAYAGGDYQATEHWS-VSLYGSRQKDAWDQYYAGTSFNYP-LDDKLSLLGGAN 245
OccD5           YPVRLDAVGFLGGQWQATERLQ-LSLYASRFDDIWQQAYFGASHRQP-LGGERALRVDLD 246
OccD6           ----LKSINVFGGKYAFTDHFN-ASLYASDVEDVLKKQYINLNYTIP-LQADQSLNFDFN 225
OccD7           G----SAIGFAGASLAASDNLS-GSLFAAQLDNVWRQVYLNMNLQR------AAFSLDGN 226
OccD8           ADRESPHIAWLGASYSAPGGSQ-ATLYSGRLEDIWNQHYLGLSQPWR-LSSQLTLTPWLH 259

OccK1           ----SDGFNYAGAEYRFNRERTQLGLWHGQLEDVYRQSYANLLHKQR-VGDW-TLGANLG 229
OccK2           ----SDRFDFAGGEYRFNGERSLLGLWNAELKDIYRQQYLQLQHSQP-LGDW-LLGANLG 232
OccK3           ----SDAFDFAGGDYRLTPALT-ASLHQGRLKDIYRQTFAGLVHTLD-LGGQRSLKSDLR 225
OccK4           ---ASDEFNLAGATYAFTPRLS-ASYYYGQLKDIYRQHYLGLLHTLP-LGEGLSLRSDLR 228
OccK5           ----ADHFDLGGLDYKLTDQLT-ASYHYSNLQDVYRQHFVGLLHSWP-IGPG-ELTSDLR 229
OccK6           SGLTSDKFDFAGGSYKWTDNLS-TSYHYGKLDNFYKQHYLGLVHTLP-IADKQSLKSDIR 235
OccK7           RHPGSDRFDFAGGTYRWSERLS-SSYHYANLEDFYRQHHLGVQHLLP-LADDQSLKSDIR 235
OccK8           D---SNKFYYAGGDYKVNKDLT-LQYYYGNLDDFYKQHFLGLIHNWQ-IGPG-VLKTDLR 238
OccK9           ----SDRFDYLGAEYRFNAERSQVGLWQARLQDIYRQDYYSLSHKQS-FGGW-RLGASVG 227
OccK10          T---GNRFDAYQLDYQVNDGLL-LQYAQGGLRNVYRQRYLGAVGKRQ-VGAG-KLSADLR 236
OccK11          Q---ADRFDLAGLDYRIAPDWT-GSYHYGELEQVYAQHFLGLKGRIG-IAAD-SLESDLR 230
                                            .   :   : .                 :

OccD1           IYRTNDE----GKAKAG--------------------DISNTTWSLAAAYTLDA-HTFTL 269
OccD2           YYKTVDS----GDSLLG--------------------RIDNNTYSLHFAVGYRQ-HTVTA 276
OccD3           GYHTGDT----GAREAG--------------------YIDNDTWSLAFTLGHRA-HALTL 313
OccD4           YYKVKDQ----GRQVMG--------------------ELDNDIWSVRGGFAYGP-HQVLL 280
OccD5           AYRTRDS----GQSRFG--------------------RIDTLTSSLALGYEHGP-QRITL 281
OccD6           GYRTKLDSDFADQNFNG--------------------NRDNKIWSLAASYTIDA-HTFMI 264
```

```
OccD7            LYRTRDQ----GRSRAG--------------------AIDTLAYSLQLKYRVGA-QGFSL 261
OccD8            YYKTRDQ----GRSQLG--------------------RIDNDLYNAGLTLAGGG-QSLSL 294

OccK1            LFVDRDD----GAARAG--------------------EIDSHTVYGLFSAGIGL-HTFYL 264
OccK2            GFRGRDA----GSARAG--------------------KLDNRTVSALFSARYGL-HTLYL 267
OccK3            FARASED----GGFR----------------------ELDNRAFGALFSLRLGA-HAVAA 258
OccK4            YFDSGED----GAAIS--------------------GPVDNRNLNAMLTLRAGA-HAFGI 263
OccK5            FARSTDS----GSAKA--------------------GGIDNKSLNGMFTYSLGN-HAFGA 264
OccK6            WARSTDD----GSS-----------------------NVDNKALNAMFTYSLGY-HAFGV 267
OccK7            WARSTDE----GGS-----------------------RVNNRALNALFTYRLGG-HAFGL 267
OccK8            AFDSSSD----GKNGSRSGRADGYVSSGYYGSGVTKGEVDNRAFSGLFTYTVSG-HSIGA 293
OccK9            LFDTRDE----GAAKLG--------------------ELENRALTGFFSATRGG-HSLGA 262
OccK10           WFDSEDA----GAARAG--------------------KIDNRALSLLLAYAQGG-HTLSA 271
OccK11           LALSRDT----GGARG--------------------GRIDNRSFSGSLTYRLRNGQAFGL 266
                            .                           ..              : .

OccD1            AYQKVHGDQPFDYIGFGRNG-SGAGGDSIFLANSV--QYSDFNGPGEKSWQARYDLNLAS 326
OccD2            VLQKVNGNTPFDYINQGD---------SIFLDNS--QQYSDFNGPNEKSWKLQYDYDFVA 325
OccD3            AYQQVDGNEYFDYVHET---------SAIFLANS---MLADYNSPNEKSAQIRYETDWSY 361
OccD4            SYQRNNGDDDFDYLRQT---------DSIYLDNS--IQYSDFNSPKERSLMLRYDLDMAA 329
OccD5            AYQRVHGEQPFDYMAFGDGR----SSASMVLANS--VGYSDFNGPGERSWQLRYDLDLGA 335
OccD6            AHQRNTGDTGYNYGWYQNAGGIGDGGTTIWLANS---YWSDFNAEDERSWQVSYALDFAK 321
OccD7            AYQRVHGDTPFDFVG-GD---------SIYLANS--IKYADFNGPGERSWQARYDLDLAP 309
OccD8            SLQKVDGDTPFDFIAQND---------RTFLYESNAMQYADFNGPGERSWKIQYQASLAF 345

OccK1            GLQKVGGDSGWQSVYG---------SSGRSMGND--MFNGNFTNADERSWQVRYDYDFVG 313
OccK2            GLQKVSGDDGWMRVNG---------TSGGTLAND--SYNASYDNPGERSWQLRYDFDFVG 316
OccK3            GYQRISGDDPYPYIAG----------SDPYLVNF--IQIGDFGNVDERSWQLRYDYDFGA 306
OccK4            GVQKMIGNDAFPVLNGY---------TTPYVANL--MAYQTFTRPQEKSWQLRYDYDFAG 312
OccK5            AWQRMNGDDAFPYLEG----------SNPYLVNF--VQVNDFAGPKERSWQLRYDYDFVG 312
OccK6            GYQKMSGDTGFAYING----------ADPYLVNF--IQIGDFANKDEKSWQARYDYNFAG 315
OccK7            GYQRMSGDSGFAYLAG----------TDPYLVNF--VQIGDFANKDERSWQLRYDYDFAA 315
OccK8            GYQILNGDSDFPFLNRGDGE-----GSTAYLITD--VQIGKFQRAGERTWQVRYGYDFAT 346
OccK9            GYQRMYGDDGMLYIAG---------TS-TPLVND--IQVRNFTSAGERSWQLRYDYDFVA 310
OccK10           GWQRMNGASSMPYLDG----------SNPYLANY--LQVNDFANPEERSWQLRYDFDLRS 319
OccK11           GYQRMSGDHGFPYLEG----------TDPYLVNF--GQYNDFAEAGESSWQLRYDCDFAP 314
                   *   *                       :          :    * :    *  .

OccD1            YGVPGLTFMVRYINGKD-------IDGTKMSDNNVGYKNYGYGEDGKHHETNLEAKYVVQ 379
OccD2            LGLPGLSASASYSRGK------LDLTRVDPDSPGYGGWYSADGKNAKHWERDLDLQYVVQ 379
OccD3            YGVPGLSTGVWYVKGWDIDG---THYDGDRNGAYGNYAEVRAQDGEKHHELGLMAAYKVQ 418
OccD4            FGVPGLSFMTRYGKGWD------ADYSNANSVYMRTDANGNPLTNQGRWERDVEVKYVVQ 383
OccD5            LGLPGLSLHALHARGR---------AGASASSAAESIYAGLYGRDGRHRENDLGFAYRVK 386
OccD6            YGVPGLTYRVAYVRGDN-------IKTAETSN-------------GKEREIFNQVQYVVQ 361
OccD7            FGLPGLSFMARYVSGRAIDGSHAPAGGAYNPLGADGRYRPLQGSGGKHWERDLDLRYLFA 369
OccD8            LAAPDWQFGAAYGRGQ------ADLTRVDPDSAGYGYLYNPNGKNAQHWERDLSLRYAFP 399

OccK1            LGWPGLIGMVRYGHGSN------ATTKAGSG--------------GKEWERDVELGYTVQ 353
OccK2            LGLPGLTFMTRYLHGDH------VRLAGVTDD-------------GSEWGRESELGYTLQ 357
OccK3            LGLPGLSFMSRYVSGDN------VARG-AAND-------------GKEWERNTDLGYVVQ 346
OccK4            LGLPGLNLMTRYVQGRD------IDRGAGRAD-------------DSEWERNTDLSYVIQ 353
OccK5            LGIPGLTFMTRYVKGDN------VELAGQSGE-------------GREWERNTELQYVFQ 353
OccK6            VGIPGLTFMTRYVKGDN------IDLLTTSGE-------------GKEWERDMDIAYVFQ 356
OccK7            IGLPGLTFMSRYLRGEH------IDLLDGGGR-------------GKEWERDTDIAYLVQ 356
OccK8            VGVPGLTFNTIYLSGD--------KIKTARGD-------------QSEWERDISLAYVIP 385
OccK9            LGIPGLTAMARYASGAH------ARTKAMDD--------------GRAWERDVDVAYVIQ 350
OccK10           VGVPGLSFMTRYVNGDH------IRLANGDE--------------GKEWERDIELKYIVQ 359
OccK11           LGVPGLSLMTRYFSGHG------AKPKGADG--------------SREWERDSDLRYVLQ 354
                                                                        * .
```

```
OccD1        SGPAKDLSFRIRQAWHRAN-----ADQG--EGDQNEFRLIVDYPLSIL- 420
OccD2        GGPAKDLSLRLRWATHRGTGGYSAVDN-----DIDEYRVIVDYPIDVF- 422
OccD3        NGPIKDSTFKLTYMMHKAS-------QNQIDGSVNELRLVSTFPFNLL- 459
OccD4        GGAAKDLAFRVRQATVRS---------DSFESDLDEVRLIVEYPLQVL- 422
OccD5        AGPLAGLALRASQAWHRGNASY--LDG-----DIDETRLVVDYSRSIW- 427
OccD6        SGPAKDLTLRLRSSFLRVS-----NDARSYNDDGNEIRAFVEYPFSVF- 404
OccD7        SGPFKDLSLNVSHLSHRANAAQ--AGD-----DIDRLYLIVEYPLKGSL 411
OccD8        AGPAKGLSVTLRWATHRPGEGYTAPGNTRGNSSSDEYRVVVDYPIRLL- 447

OccK1        SGPLARLNVRLNHASNRR--------SFNS--DFDQTRLVVSYPLSW-- 390
OccK2        SGAFKRLNVRWRNSSQRR--------DWGSNTRFDENRLIVSYPLSLL- 397
OccK3        SGPLKNLGVKWRNATVRS----------NFANDLDENRLILSYSLALW- 384
OccK4        SGPLKSVALKWRNITYRS----------RYGADLDENRFIVNYTLKLW- 391
OccK5        SGALKNLGIRWRNATFRS----------NFTRDIDENRLIVSYTLPIW- 391
OccK6        SGPLKNLGVKWRNATMRT----------NYTNDYDENRLIVSYTLPLW- 394
OccK7        SGPLKNLGIKLRNGTFRS----------DFGNDIDETRLIVSYALPLW- 394
OccK8        DGTFKGLGFTWKNASFRSGLPA--AGSSNNQRDQDENRLIVSYTLPLL- 431
OccK9        SGPLKNLGLRWRNAMLRS--------NHAA--DVDENRLILSYSLPLL- 388
OccK10       SGRFKDLSLRLRNATYRT-------DFERSARDVDEVRLIASYNLSLF- 400
OccK11       GGALKGLGLVWRNATYRS----------AFSRDIDENRLYLTYELPLF- 392
              *       .       :                 :.      :
```

Figure S1. CLUSTALW multiple sequence alignment of OccD/OccK channels. Invariant residues (*), highly conserved residues (:) and conserved residues (.) are shown at the bottom. The observed secondary structure elements for OccD1 and OccK1 are shown (β strands colored as blue arrows for OccD1 and as salmon arrows for OccK1; α-helices in red). The missing residues in the crystal structures are shown as hatched orange bars. The residues that line the pore constriction are highlighted in green. Amino acid residues that are part of the basic ladder are highlighted in pink. Pore lining basic residues that are also part of the basic ladder are highlighted in purple. The L7 insertion for OccD1 is highlighted in yellow and the N-terminal extension of OccD3 is shown in cyan.
