# Supplementary material for: Substrate Specificity within a Family of Outer Membrane Carboxylate Channels
Source: PLoS Biol. 2012 Jan 17;10(1):e1001242. doi: 10.1371/journal.pbio.1001242 (PMC3260308; doi:10.1371/journal.pbio.1001242)
Supplement: Figure S2 — Variable pore openings for OccD channels. (A) The small OccD3 pore as present in the crystal structure and (B) the large pore that could potentially be formed after movement of the N-terminal extension (shown in red; residues 1–30), for example as a result of high ionic strength in the single channel conductance experiments. (C) The small pore in the current structure of OccD1. Note the different conformation of the insertion in loop L7 (colored green) compared to that in the original OccD1 structure (PDB ID: 2ODJ). As a consequence, OccD1 in the original structure has a larger pore (D). In all cases, the view is from the extracellular side. (PDF) [file pbio.1001242.s002.pdf]

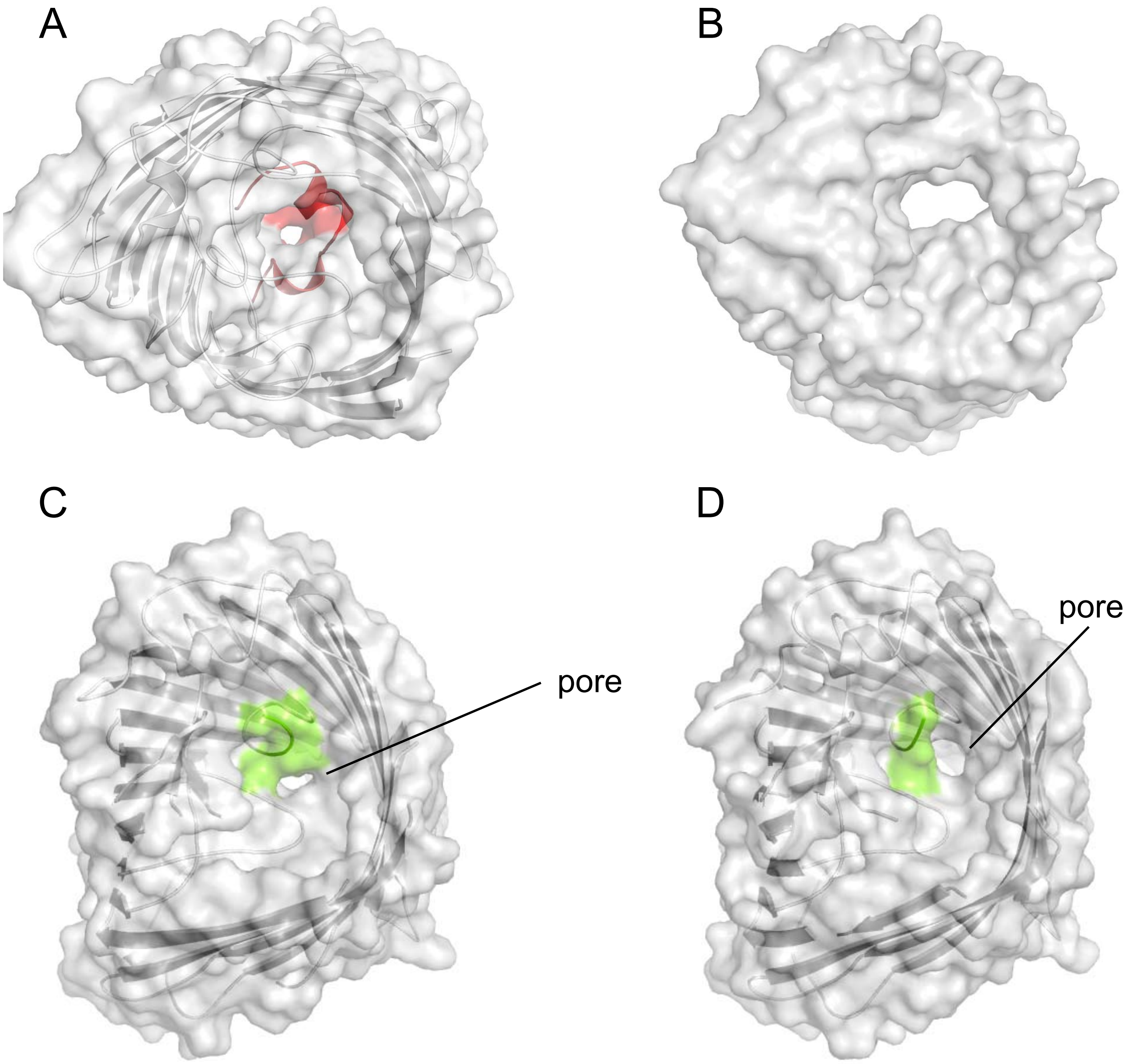

Figure S2. Variable pore openings for OccD channels. (A) The small OccD3 pore as present in the crystal structure and (B) the large pore that could potentially be formed after movement of the N-terminal extension (shown in red; residues 1-30), for example as a result of high ionic strength in the single channel conductance experiments. (C) The small pore in the current structure of OccD1. Note the different conformation of the insertion in loop L7 (colored green) compared to that in the original OccD1 structure (PDB ID: 2ODJ). As a consequence, OccD1 in the original structure has a larger pore (D). In all cases the view is from the extracellular side.
