# Supplementary material for: Substrate Specificity within a Family of Outer Membrane Carboxylate Channels
Source: PLoS Biol. 2012 Jan 17;10(1):e1001242. doi: 10.1371/journal.pbio.1001242 (PMC3260308; doi:10.1371/journal.pbio.1001242)
Supplement: Figure S3 — Occ channels have differently sized shape and pores. (A) Wire diagrams from the side made with HOLE [1], showing the surface of the Occ channel pores in blue. Areas of the pores with a diameter smaller than 4.5 Å are colored green. The extracellular side is at the top. The pores for OccD1 and OccD2 could not be calculated. (B) Pore radii plotted as a function of the pore coordinate z. Channels were aligned to OccD3 and radii calculated using HOLE. Due to the complex pore shape and absence of a pore for OccD1 and OccD2, respectively, profiles for these two channels could not be calculated. (PDF) [file pbio.1001242.s003.pdf]

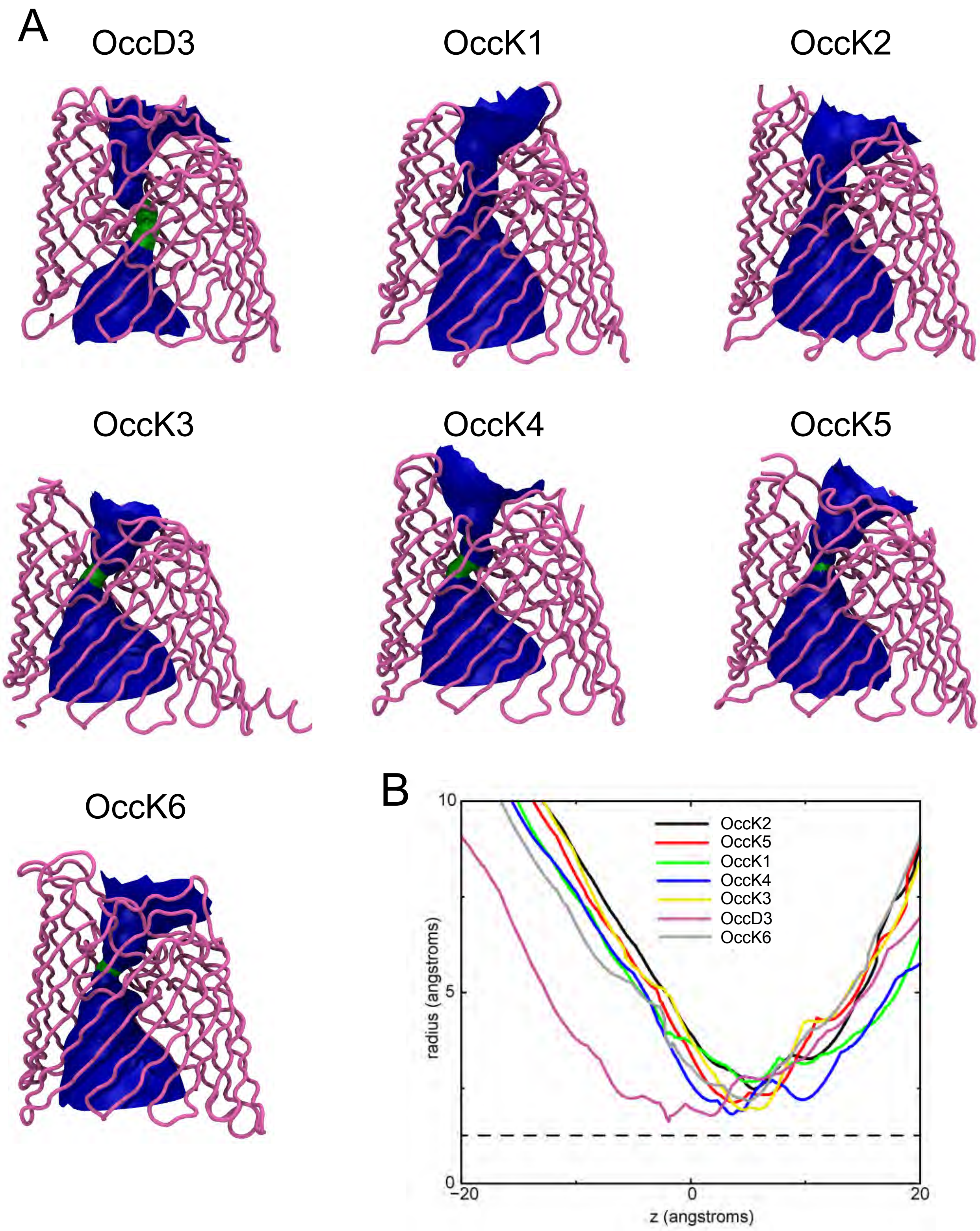

Figure S3. Occ channels have differently sized shape and pores. (A) Wire diagrams from the side made with HOLE [1], showing the surface of the Occ channel pores in blue. Areas of the pores with a diameter smaller than 4.5 Å are colored green. The extracellular side is at the top. The pores for OccD1 and OccD2 could not be calculated. (B) Pore radii plotted as a function of the pore coordinate z. Channels were aligned to OccD3 and radii calculated using HOLE. Due to the complex pore shape and absence of a pore for OccD1 and OccD2 respectively, profiles for these two channels could not be calculated.
