# Supplementary material for: Substrate Specificity within a Family of Outer Membrane Carboxylate Channels
Source: PLoS Biol. 2012 Jan 17;10(1):e1001242. doi: 10.1371/journal.pbio.1001242 (PMC3260308; doi:10.1371/journal.pbio.1001242)
Supplement: Figure S4 — Single-channel electrical recordings of (A) OccD4, (B) OccD5, (C) OccD6, and (D) OccK7. The data were collected at an applied transmembrane potential of +80 mV. The buffer solution in the chamber contained 1 M KCl, 10 mM potassium phosphate, pH = 7.4. For the sake of clarity, the single-channel electrical traces were low-pass Bessel filtered at 2 kHz. The numbers located above the traces represent the single-channel conductance of the most probable sub-state of the channel. The averages were derived from at least three independent single-channel electrical recordings. (PDF) [file pbio.1001242.s004.pdf]

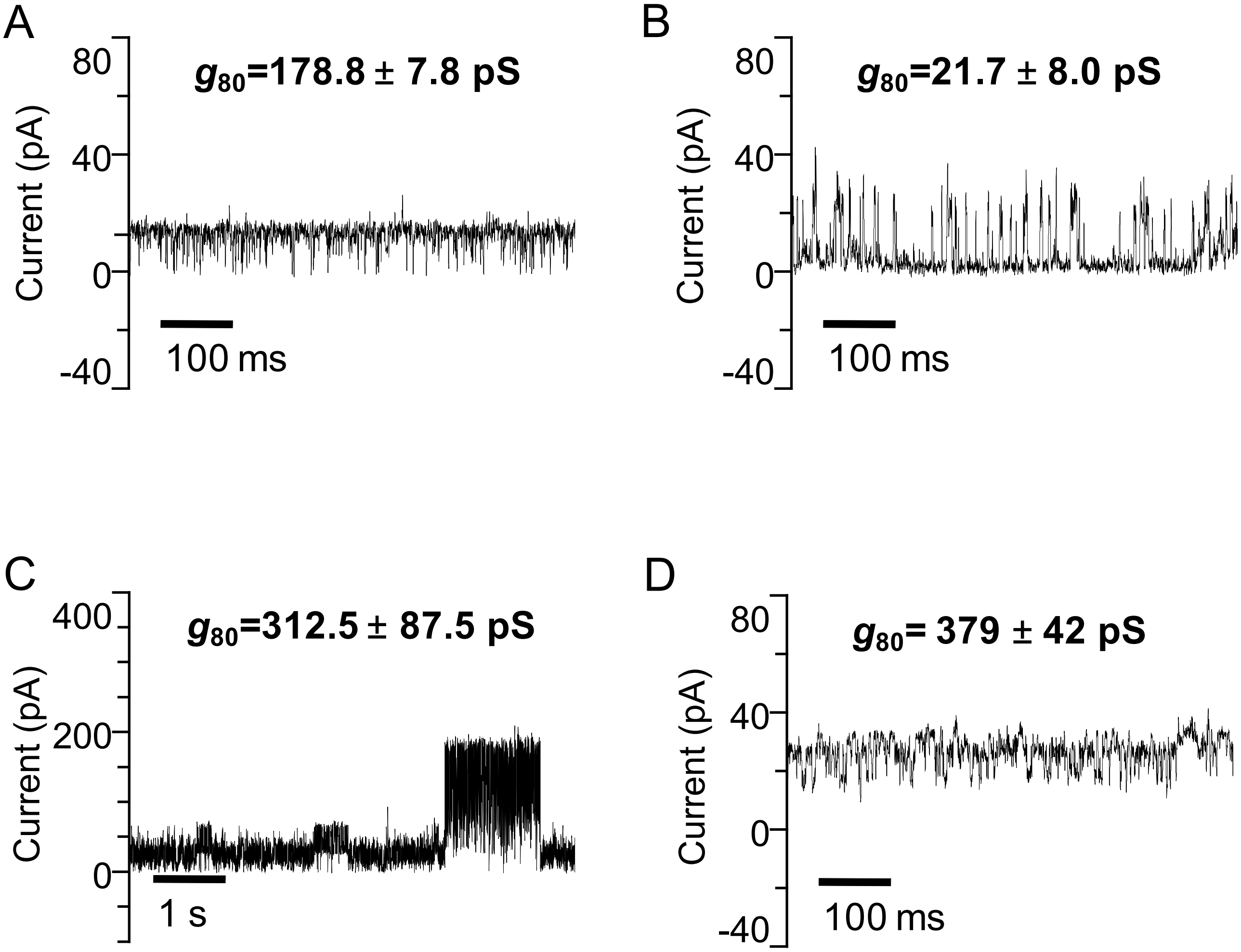

Figure S4. Single-channel electrical recordings of (A) OccD4, (B) OccD5, (C) OccD6 and (D) OccK7. The data were collected at an applied transmembrane potential of +80 mV. The buffer solution in the chamber contained 1 M KCl, 10 mM potassium phosphate, pH=7.4. For the sake of clarity, the single-channel electrical traces were low-pass Bessel filtered at 2kHz. The numbers located above the traces represent the single-channel conductance of the most probable sub-state of the channel. The averages were derived from at least three independent single-channel electrical recordings.
