# Supplementary material for: Substrate Specificity within a Family of Outer Membrane Carboxylate Channels
Source: PLoS Biol. 2012 Jan 17;10(1):e1001242. doi: 10.1371/journal.pbio.1001242 (PMC3260308; doi:10.1371/journal.pbio.1001242)
Supplement: Figure S5 — Comparison of 3H-arginine uptake in OccD1 proteoliposomes made using either E. coli or P. putida lipids. 0.25 µM radiolabeled arginine was added to proteoliposomes and the transport reaction was stopped after 15 min. 3H-arginine uptake in P. putida proteoliposomes containing internal E. coli LAO-binding protein (Lysine-Arginine-Ornithine binding protein) is also shown. LAO-binding protein inside the proteoliposomes potentially forms a “sink” for arginine transport. (PDF) [file pbio.1001242.s005.pdf]

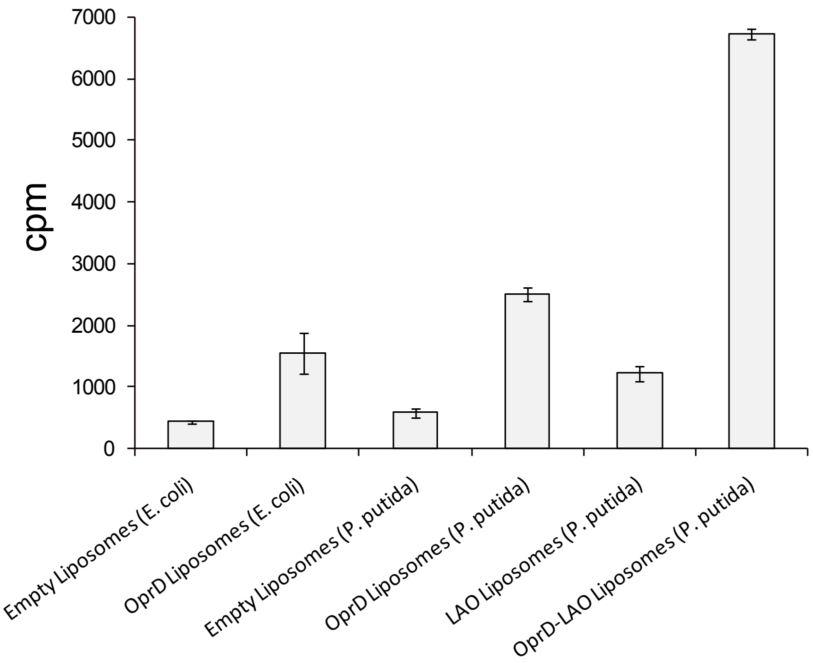

Figure S5. Comparison of ^{3}H-arginine uptake in OccD1 proteoliposomes made using either *E. coli* or *P. putida* lipids. 0.25 μM radiolabeled arginine was added to proteoliposomes and the transport reaction was stopped after 15 minutes. ^{3}H-arginine uptake in *P. putida* proteoliposomes containing internal *E. coli* LAO-binding protein (Lysine-Arginine-Ornithine binding protein) is also shown. LAO-binding protein inside the proteoliposomes potentially forms a “sink” for arginine transport.
