# Supplementary material for: Substrate Specificity within a Family of Outer Membrane Carboxylate Channels
Source: PLoS Biol. 2012 Jan 17;10(1):e1001242. doi: 10.1371/journal.pbio.1001242 (PMC3260308; doi:10.1371/journal.pbio.1001242)
Supplement: Figure S6 — Expression levels of channels in total membrane vesicles. Representative Western blots of vesicles containing Occ channels, FadL, and OmpG: (1) OccD4; (2) OccK1; (3) OccD2; (4) OccK2; (5) OccD1; (6) Pure OccD1 (Control); (7) OccK5; (8) OccD5; (9) OccK4; (10) OccK3; (11) OccD3; (12) OccD6; (13) OccK7; (14) OccK6; (15) Pure OccD1 (Control); (16) OmpG; and (17) FadL. Vesicles were diluted to contain 2 µg total membrane protein and the band intensity from Occ channels was compared to that of 0.1 µg purified OccD1 (control). (PDF) [file pbio.1001242.s006.pdf]

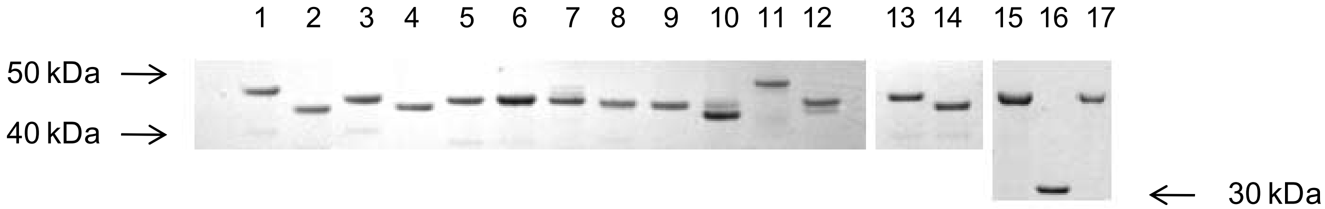

Figure S6. Expression levels of channels in total membrane vesicles. Representative western blots of vesicles containing Occ channels, FadL and OmpG: (1) OccD4; (2) OccK1; (3) OccD2; (4) OccK2; (5) OccD1; (6) Pure OccD1 (Control); (7) OccK5; (8) OccD5; (9) OccK4; (10) OccK3; (11) OccD3; (12) OccD6; (13) OccK7; (14) OccK6; (15) Pure OccD1 (Control); (16) OmpG; and (17) FadL. Vesicles were diluted to contain 2 μg total membrane protein and the band intensity from Occ channels were compared to that of 0.1 μg purified OccD1 (control).
