# Supplementary material for: Substrate Specificity within a Family of Outer Membrane Carboxylate Channels
Source: PLoS Biol. 2012 Jan 17;10(1):e1001242. doi: 10.1371/journal.pbio.1001242 (PMC3260308; doi:10.1371/journal.pbio.1001242)
Supplement: Figure S7 — Right-side-out orientation of Occ channels in membrane vesicles. (A) Effect of freeze-thaw cycles on specific substrate transport activity (0.25 µM 3H-arginine, 15-min assay by OccD1 vesicles). The specific activity decreases upon freeze-thawing, presumably as a result of random reorientation of OccD1 channels in the vesicles. (B) Detection of the OccD1 N-terminal histidine tag by anti-His antibody. The tag, originally inaccessible to the antibody (i.e., lumenal), becomes more accessible upon freezing-thawing. (PDF) [file pbio.1001242.s007.pdf]

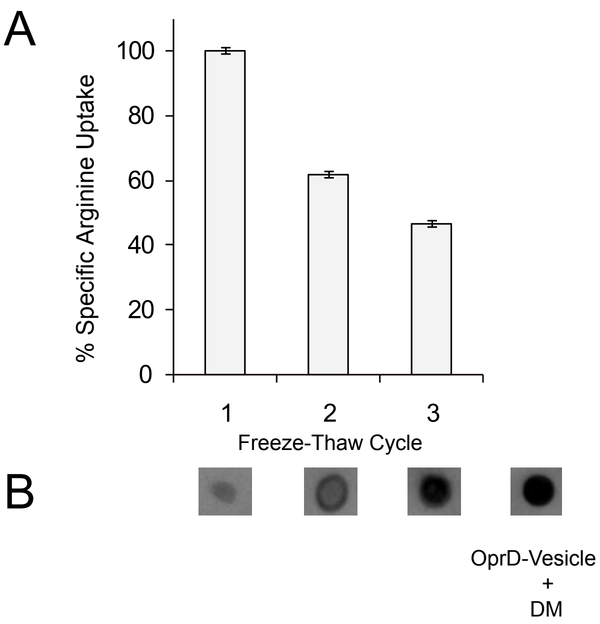

Figure S7. Right-side-out orientation of Occ channels in membrane vesicles. (A) Effect of freeze-thaw cycles on specific substrate transport activity (0.25 μM ^{3}H-arginine, 15 minute assay by OccD1 vesicles). The specific activity decreases upon freeze-thawing, presumably as a result of random reorientation of OccD1 channels in the vesicles. (B) Detection of the OccD1 N-terminal histidine tag by anti-His antibody. The tag, originally inaccessible to the antibody (*i.e.* lumenal), becomes more accessible upon freezing-thawing.
