# Supplementary material for: Substrate Specificity within a Family of Outer Membrane Carboxylate Channels
Source: PLoS Biol. 2012 Jan 17;10(1):e1001242. doi: 10.1371/journal.pbio.1001242 (PMC3260308; doi:10.1371/journal.pbio.1001242)
Supplement: Figure S8 — Optimization of substrate uptake by Occ channels in total membrane vesicles relative to “empty” background (pB22). Assays contain varying concentrations of radiolabeled substrates (0.05–10 µM) as indicated in the figure. Uptake times were as follows: arginine, 15 min; benzoate, 10 min; glucuronate, 10 min; pyroglutamate, 15 min; vanillate, 40 min; glucose, 15 min; citrate, 40 min; and phenylacetate, 40 min. (PDF) [file pbio.1001242.s008.pdf]

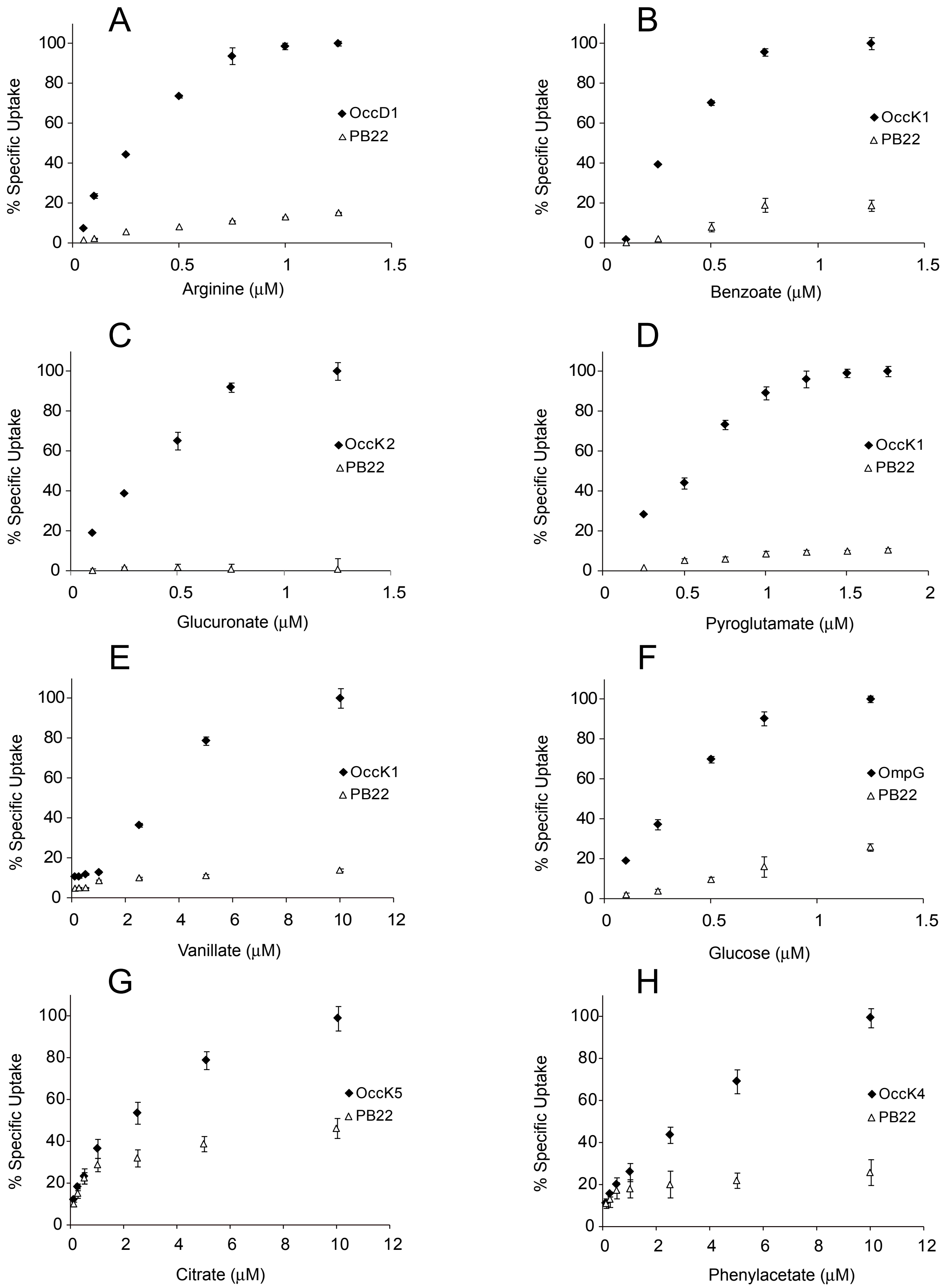

Figure S8. Optimization of substrate uptake by Occ channels in total membrane vesicles relative to "empty" background (pB22). Assays contain varying concentrations of radiolabeled substrates (0.05 μM-10μM) as indicated in the figure. Uptake times were as follows: arginine, 15 minutes; benzoate, 10 minutes; glucuronate, 10 minutes; pyroglutamate, 15 minutes; vanillate, 40 minutes; glucose, 15 minutes; citrate, 40 minutes and, phenylacetate, 40 minutes.
