# Supplementary material for: Substrate Specificity within a Family of Outer Membrane Carboxylate Channels
Source: PLoS Biol. 2012 Jan 17;10(1):e1001242. doi: 10.1371/journal.pbio.1001242 (PMC3260308; doi:10.1371/journal.pbio.1001242)
Supplement: Figure S9 — Time curves of arginine uptake by OccD1 at varying concentrations of substrate. Arginine was added to the uptake assays at the final concentrations indicated in the figure and samples were taken at 2, 5, 10, 20, and 30 min. (PDF) [file pbio.1001242.s009.pdf]

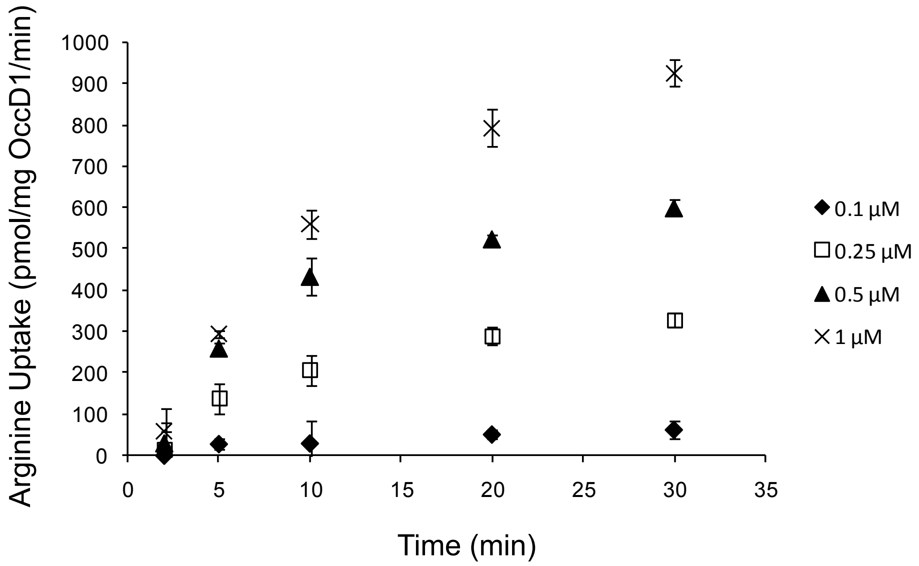

Figure S9. Time curves of arginine uptake by OccD1 at varying concentrations of substrate. Arginine was added to the uptake assays at the final concentrations indicated in the figure and samples were taken at 2, 5, 10, 20 and 30 minutes.
