# Supplementary material for: Substrate Specificity within a Family of Outer Membrane Carboxylate Channels
Source: PLoS Biol. 2012 Jan 17;10(1):e1001242. doi: 10.1371/journal.pbio.1001242 (PMC3260308; doi:10.1371/journal.pbio.1001242)
Supplement: Figure S10 — Structures of (A) radiolabeled Occ channel substrates and (B) antibiotics used for uptake competition experiments in this study. Compounds that do not contain a carboxyl group are labeled in red. (PDF) [file pbio.1001242.s010.pdf]

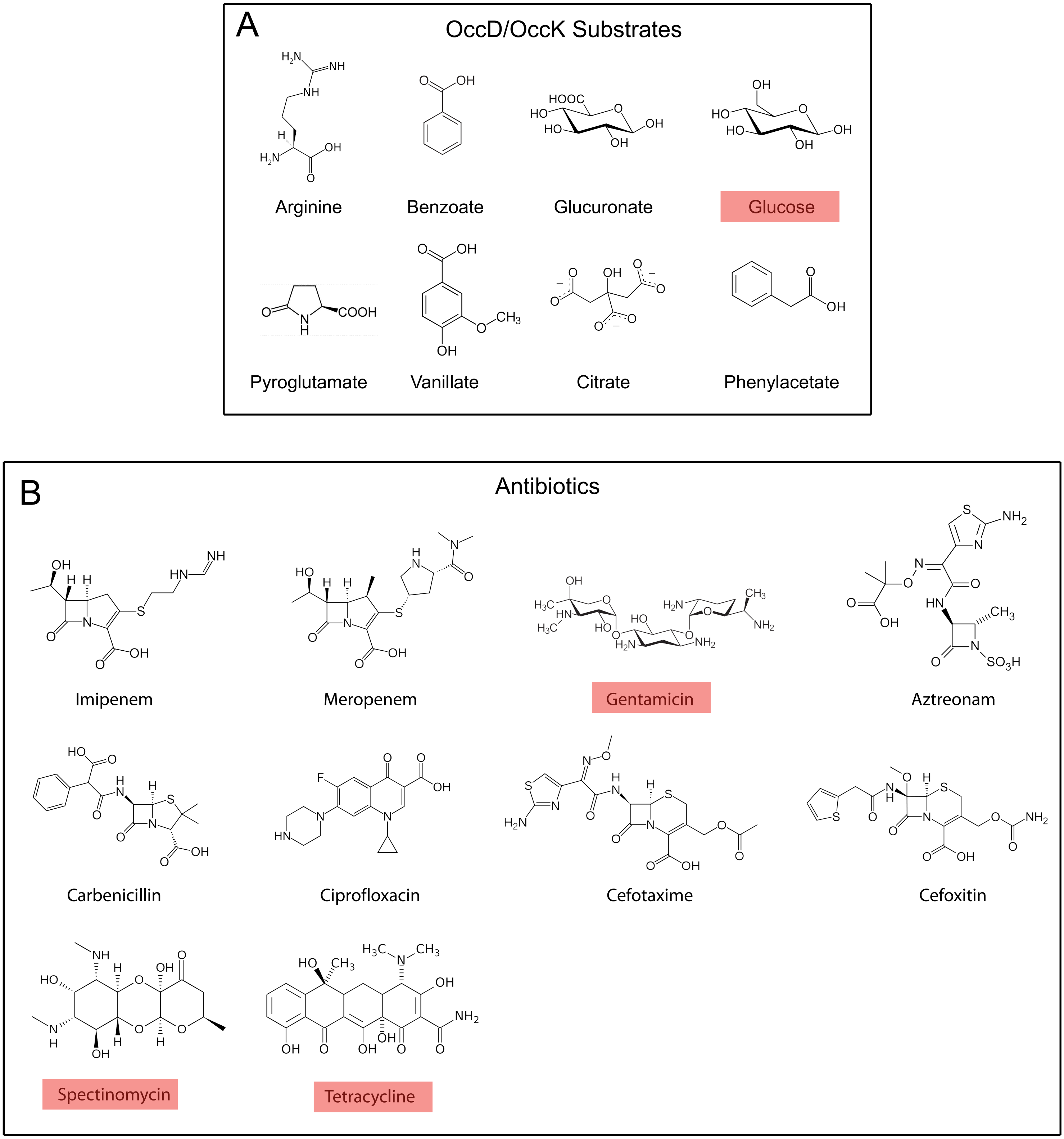

Figure S10. Structures of (A) radiolabeled Occ channel substrates and (B) antibiotics used for uptake competition experiments in this study. Compounds that do not contain a carboxyl group are labeled in red.
