# Supplementary material for: Substrate Specificity within a Family of Outer Membrane Carboxylate Channels
Source: PLoS Biol. 2012 Jan 17;10(1):e1001242. doi: 10.1371/journal.pbio.1001242 (PMC3260308; doi:10.1371/journal.pbio.1001242)
Supplement: Figure S11 — Uptake of non-preferred radiolabeled substrates in E. coli Bl21 omp8 total membrane vesicles, expressing empty plasmid (pB22), Occ channels, or E. coli OmpG or FadL. Substrates are (A) vanillate (10 µM, 45 min), (B) phenylacetate (10 µM, 45 min), and (C) citrate (10 µM, 45 min). 100% specific activities correspond to 27.8±1.7 (A), 7.6±0.5 (B), and, 6.2±0.6 (C) pmoles substrate/min/mg protein. (PDF) [file pbio.1001242.s011.pdf]

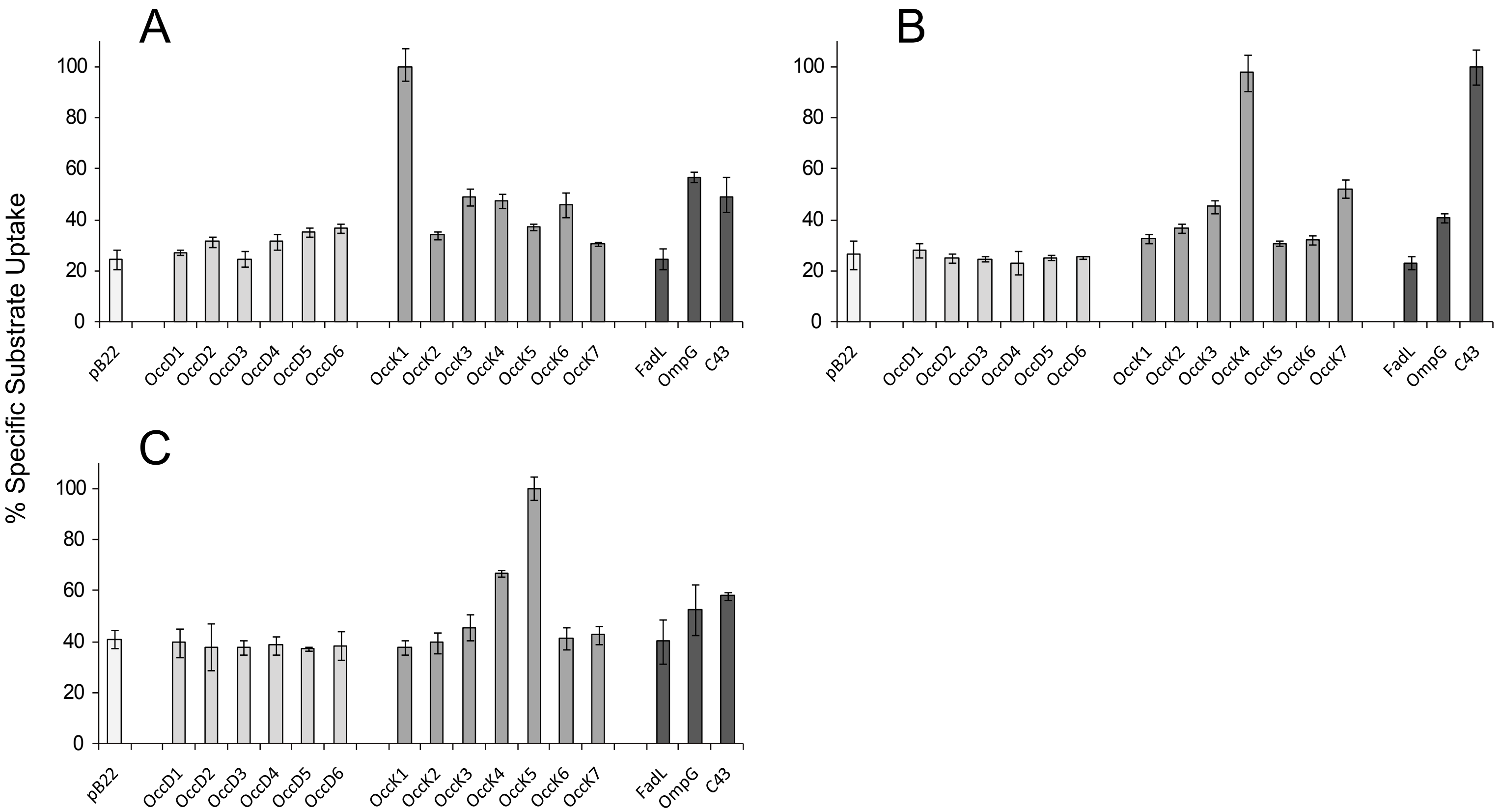


**Figure S11.** Uptake of non-preferred radiolabeled substrates in *E. coli* Bl21 omp8 total membrane vesicles, expressing empty plasmid (pB22), Occ channels or *E. coli* OmpG or FadL. Substrates are (A) vanillate (10 μM, 45 minutes), (B) phenylacetate (10 μM, 45 minutes) and, (C) citrate (10 μM, 45 minutes). 100% specific activities correspond to 27.8 ± 1.7 (A), 7.6 ± 0.5 (B) and, 6.2 ± 0.6 (**C**) pmoles substrate/min/mg protein.
