# Supplementary material for: Substrate Specificity within a Family of Outer Membrane Carboxylate Channels
Source: PLoS Biol. 2012 Jan 17;10(1):e1001242. doi: 10.1371/journal.pbio.1001242 (PMC3260308; doi:10.1371/journal.pbio.1001242)
Supplement: Figure S12 — Vanillate binding sites in the OccK1 structure. (A) Cartoon overview from the side, showing the two vanillate molecules as stick models (carbons, yellow; oxygens, red). For orientation, the central basic ladder residue Arg381 lining the pore constriction is also shown. The extracellular side is at the top of the figure. (B) Stereo diagram showing the binding pocket for the periplasmic vanillate molecule (VAN 2). Electron density (2Fo–Fc map, contoured at 1.5 σ) is shown as a blue mesh. (PDF) [file pbio.1001242.s012.pdf]

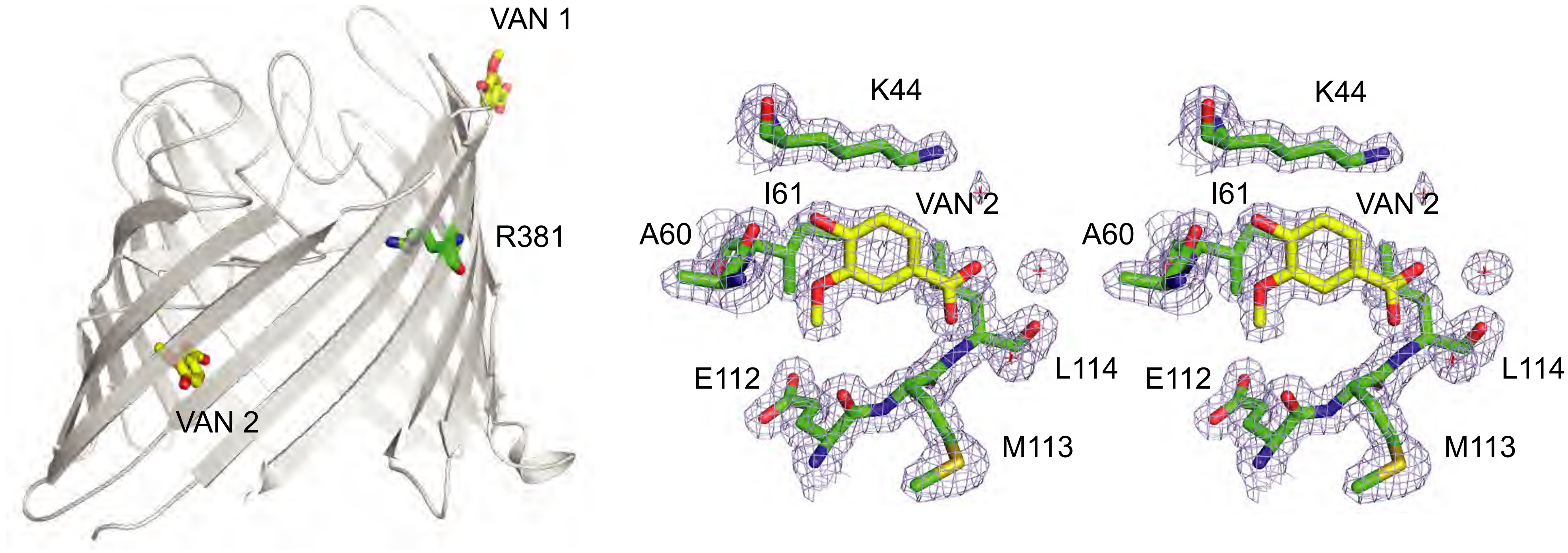

Figure S12. Vanillate binding sites in the OccK1 structure. (A) Cartoon overview from the side, showing the two vanillate molecules as stick models (carbons yellow, oxygens red). For orientation, the central basic ladder residue Arg381 lining the pore constriction is also shown. The extracellular side is at the top of the figure. (B) Stereo diagram showing the binding pocket for the periplasmic vanillate molecule (VAN 2). Electron density ($2F_o$-F_c map, contoured at 1.5σ) is shown as a blue mesh.
