# Supplementary material for: Substrate Specificity within a Family of Outer Membrane Carboxylate Channels
Source: PLoS Biol. 2012 Jan 17;10(1):e1001242. doi: 10.1371/journal.pbio.1001242 (PMC3260308; doi:10.1371/journal.pbio.1001242)
Supplement: Figure S13 — Antibiotic transport by Occ channels. Arginine (A–B) and benzoate (C–F) uptake measured in the presence of a 10-fold excess of antibiotics. The following channels are shown: OccD5 (A), OccD6 (B), OccK4 (C), OccK5 (D), OccK6 (E), and OccK7 (F). (PDF) [file pbio.1001242.s013.pdf]

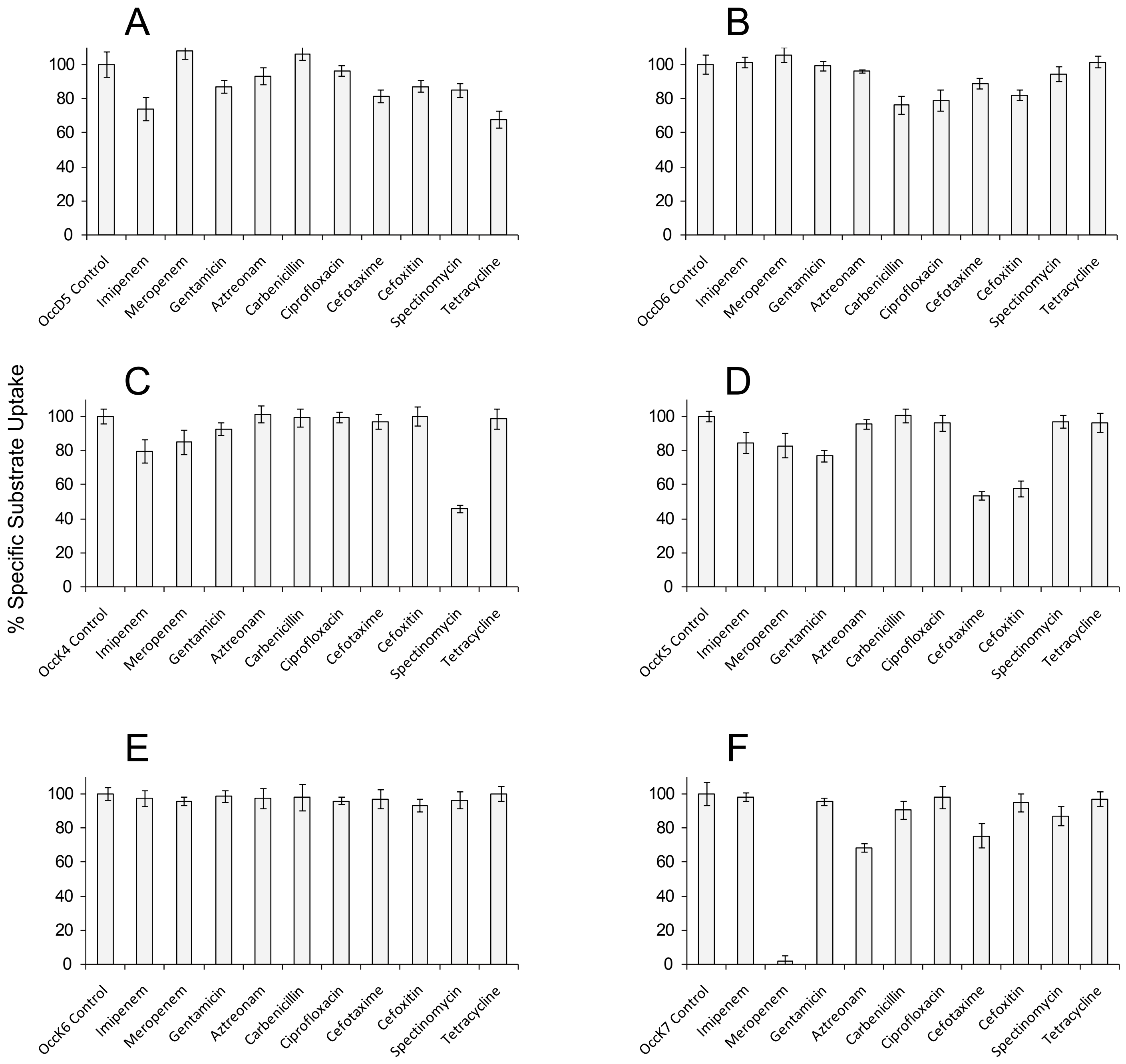

Figure S13. Antibiotic transport by Occ channels. Arginine (A-B) and benzoate (C-F) uptake measured in the presence of a 10-fold excess of antibiotics. The following channels are shown: OccD5 (A), OccD6 (B), OccK4 (C), OccK5 (D), OccK6 (E) and OccK7 (F).
