# Supplementary material for: Substrate Specificity within a Family of Outer Membrane Carboxylate Channels
Source: PLoS Biol. 2012 Jan 17;10(1):e1001242. doi: 10.1371/journal.pbio.1001242 (PMC3260308; doi:10.1371/journal.pbio.1001242)
Supplement: Figure S14 — Effect of vesicle permeabilization on arginine transport. (A) Arginine uptake by OccD1 vesicles in the presence of varying concentrations of polymyxin B. 100% corresponds to arginine uptake measured with OccD1 control vesicles that were not incubated with polymyxin B. (B) Amount of vesicles remaining on the filter as measured by total protein quantification of OccD1 control vesicles (100%) and OccD1 vesicles incubated with 0.5% polymyxin B. (PDF) [file pbio.1001242.s014.pdf]

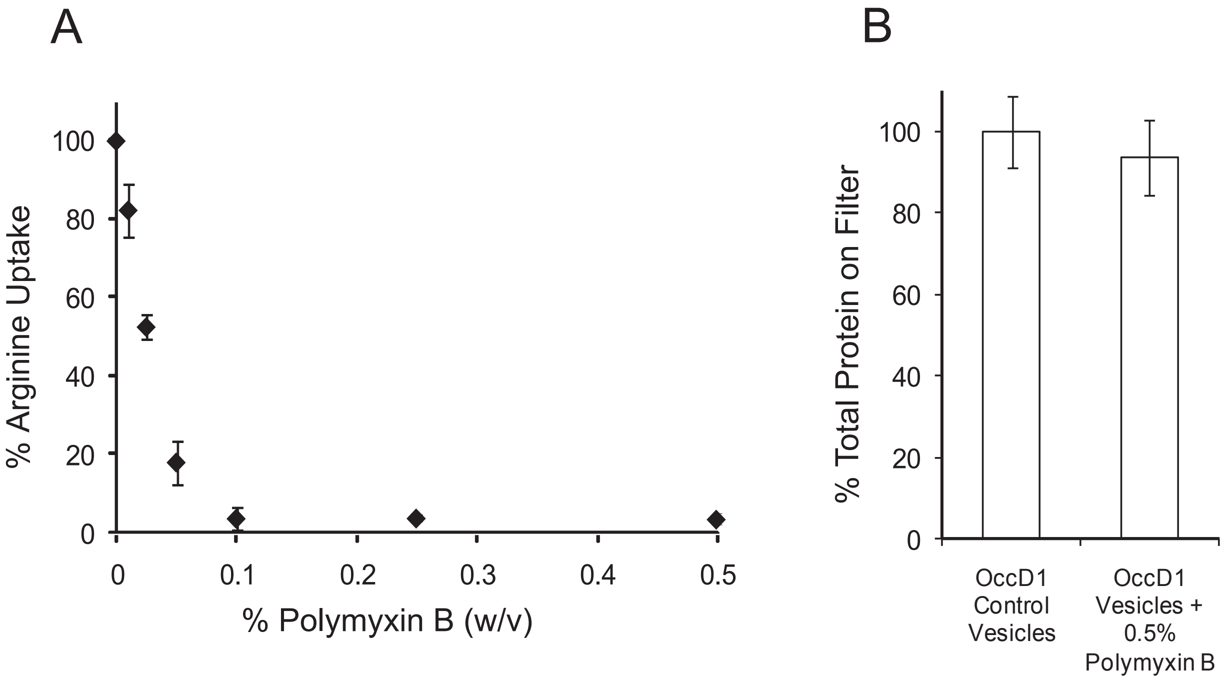

Figure S14. Effect of vesicle permeabilization on arginine transport. (A) Arginine uptake by OccD1 vesicles in the presence of varying concentrations of polymyxin B. 100% corresponds to arginine uptake measured with OccD1 control vesicles that were not incubated with polymyxin B. (B) Amount of vesicles remaining on the filter as measured by total protein quantification of OccD1 control vesicles (100%) and OccD1 vesicles incubated with 0.5% polymyxin B.
