# Supplementary material for: Substrate Specificity within a Family of Outer Membrane Carboxylate Channels
Source: PLoS Biol. 2012 Jan 17;10(1):e1001242. doi: 10.1371/journal.pbio.1001242 (PMC3260308; doi:10.1371/journal.pbio.1001242)
Supplement: Figure S15 — Backbone cartoon views of OccD1 and OccK1 viewed from the extracellular side colored by B-factor (blue, low B-factors; red, high B-factors). Note the high B-factors for the L3 loop and L7 insertion (L7i) for OccD1 relative to the barrel wall, indicating possible mobility of these segments. (PDF) [file pbio.1001242.s015.pdf]

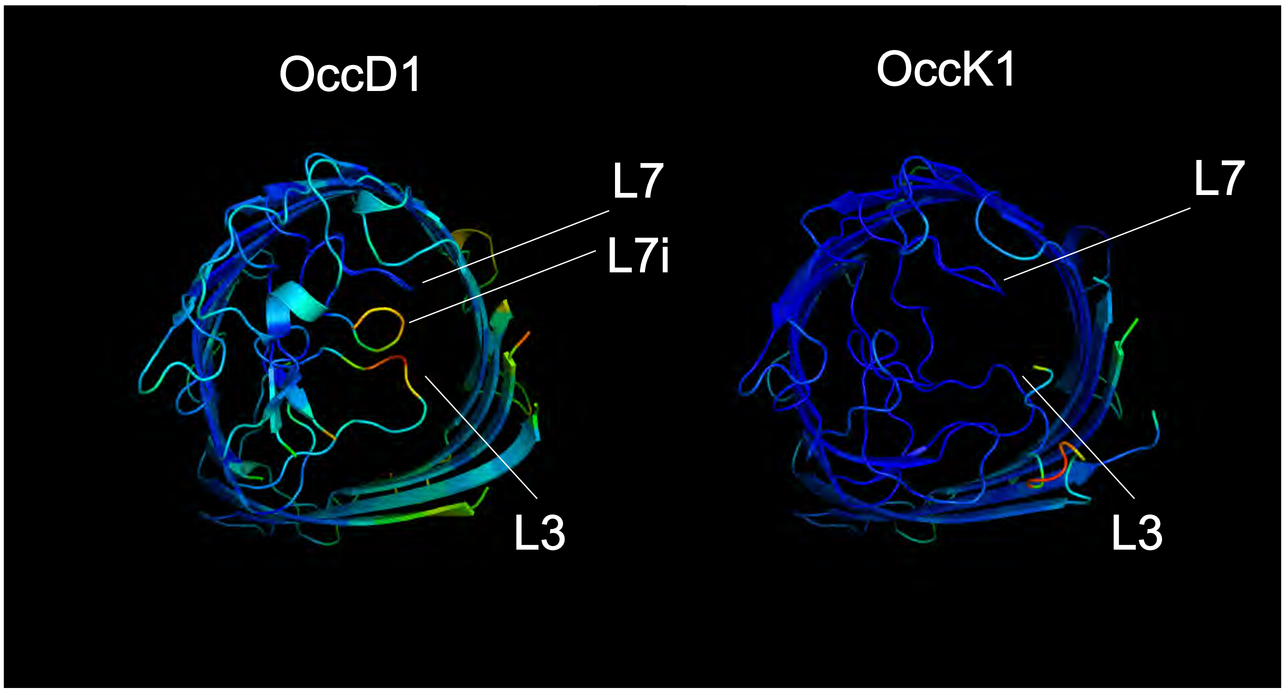

Figure S15. Backbone cartoon views of OccD1 and OccK1 viewed from the extracellular side colored by B-factor (blue; low B-factors, red; high B-factors). Note the high B-factors for the L3 loop and L7 insertion (L7i) for OccD1 relative to the barrel wall, indicating possible mobility of these segments.
