# Supplementary material for: Substrate Specificity within a Family of Outer Membrane Carboxylate Channels
Source: PLoS Biol. 2012 Jan 17;10(1):e1001242. doi: 10.1371/journal.pbio.1001242 (PMC3260308; doi:10.1371/journal.pbio.1001242)
Supplement: Table S1 — Proposed nomenclature change for Occ channels. (PDF) [file pbio.1001242.s016.pdf]

Table S1. Proposed nomenclature change for Occ channels

Old Name	New Name
**OccD subfamily**	
OprD	***OccD1***[a]
OpdC	***OccD2***
OpdP	***OccD3***
OpdT	*OccD4*[b]
OpdI	*OccD5*
OprQ	*OccD6*
OpdB	OccD7
OpdJ	OccD8
**OccK subfamily**	
OpdK	***OccK1***
OpdF	***OccK2***
OpdO	***OccK3***
OpdL	***OccK4***
OpdH	***OccK5***
OpdQ	***OccK6***
OpdD	*OccK7*
OprE	OccK8
OpdG	OccK9
OpdN	OccK10
OpdR	OccK11

[a]Channels listed in bold italics have solved crystal structures

[b]Channels listed in italics have been characterized in the current study
