# Supplementary material for: Substrate Specificity within a Family of Outer Membrane Carboxylate Channels
Source: PLoS Biol. 2012 Jan 17;10(1):e1001242. doi: 10.1371/journal.pbio.1001242 (PMC3260308; doi:10.1371/journal.pbio.1001242)
Supplement: Table S2 — Data collection and refinement statistics of Occ channels. (PDF) [file pbio.1001242.s017.pdf]

Table S2. Data collection and refinement statistics of OccD channels

		OccD1	OccD2	OccD3
**Data Collection**				
Beamline		NSLS X25	NSLS X6A	NSLS X6A
Wavelength		1.10	0.972	0.972
Space Group		C2	P1	$C222_1$
Cell Dimensions				
	a,b,c (Å)	136.4, 103.9, 47.5	93.4, 102.8, 101.7	99.9, 159.8, 82.1
	α,β,γ (˚)	90, 107.7, 90	77.7, 62.6, 62.9	90,90,90
Resolution (Å)		50-2.15	40-2.8	40.2.7
Completeness[1]		98 (80.8)	98.3 (98.1)	99.8 (100.0)
Redundancy		4.0 (2.7)	2.6 (2.6)	7.2 (7.1)
I/σ		25.0 (2.7)	19.5 (2.8)	25.6 (3.4)
R_{sym}		5.6 (33.0)	5.6 (45.3)	9.4 (66.4)
**Refinement**				
Resolution (Å)		20-2.15	15-2.8	10-2.7
Unique Reflections[2]		33514 (2001)	71573 (1983)	18004 (1802)
R_{work}/R_{free}[3]		19.6/22.6	21.7/26.6	21.4/26.7
R.m.s.d.				
	bond lengths (Å)	0.007	1.08	0.008
	bond angles (˚)	1.07	1.30	1.16
Average B-factors				
	protein/detergent/water	41/72/43	69/77/54	49/54/45
Ramachandran				
	favored/outliers (%)	96.0/0.0	95.8/0.1	95.0/0.2
Molprobity Clashscore		6.6	28.7	21.1

[1]Values in parentheses are for the highest resolution shell

[2]Values in parentheses are the number of reflections used to calculate R_{free}

[3]$R_{work} = \sum|Fo–Fc|/\sum Fo$. R_{free} is the cross-validation of the R-factor

Table S2 continued. Data collection and refinement statistics of OccK channels

		OccK1	OccK2	OccK3
**Data Collection**				
Beamline		NSLS X25	NSLS X6A	NSLS X6A
Wavelength		1.10	0.972	0.976
Space Group		$P2_12_12_1$	$P2_1$	C2
Cell Dimensions				
	a,b,c (Å)	82.1, 96.8, 121.6	46.3, 206.9, 51.6	148.9, 78.8, 50.5
	α,β,γ (˚)	90, 90, 90	90, 99.1, 90	90, 91.6, 90
Resolution (Å)		50-1.65	50-2.3	50-1.4
Completeness[1]		99.8 (99.9)	92.3 (78.4)	99.4 (99.0)
Redundancy		7.7 (5.7)	3.6 (3.4)	3.7 (3.6)
I/σ		44.8 (2.9)	15.5 (3.0)	34.9 (1.9)
R_{sym}		6.1 (59.0)	9.8 (44.0)	4.8 (77.2)
**Refinement**				
Resolution (Å)		20-1.65	15-2.3	15-1.45
Unique Reflections[2]		113,038 (1945)	38,591 (2241)	102,362 (7858)
R_{work}/R_{free}[3]		18.0/20.1	22.5/28.1	18.3/19.8
R.m.s.d.				
	bond lengths (Å)	0.006	0.008	0.006
	bond angles (˚)	1.06	1.19	1.10
Average B-factors				
	protein/detergent/water	25/51/39	27/31/27	23/50/39
Ramachandran				
	favored/outliers (%)	97.0/0.1	96.3/0.4	97.2/0.3
Molprobity Clashscore		11.7	22.1	9.5

[1]Values in parentheses are for the highest resolution shell

[2]Values in parentheses are the number of reflections used to calculate R_{free}

[3]$R_{work} = \sum|Fo–Fc|/\sum Fo$. R_{free} is the cross-validation of the R-factor

Table S2 continued. Data collection and refinement statistics of OccK channels

		OccK4	OccK5	OccK6
**Data Collection**				
Beamline		NSLS X6A	NSLS X6A	NSLS X6A
Wavelength		0.979	1.00	0.972
Space Group		$C222_1$	$C222_1$	P1
Cell Dimensions				
	a,b,c (Å)	91.2, 115.1, 100.6	86.1, 124.0, 114.3	64.7, 78.5, 97.5
	α,β,γ (˚)	90, 90, 90	90, 90, 90	77.8, 81.5, 70.0
Resolution (Å)		40-2.1	50-2.6	50-2.35
Completeness[1]		95.1 (97.0)	99.9 (100.0)	98.2 (97.3)
Redundancy		5.1 (5.0)	7.3 (6.7)	2.8 (2.6)
I/σ		23.5 (2.3)	26.2 (2.6)	17.7 (1.8)
R_{sym}		7.9 (66.7)	11.3 (74.0)	8.4 (49.4)
**Refinement**				
Resolution (Å)		20-2.2	15-2.6	15-2.4
Unique Reflections[2]		24,521 (1289)	19,128 (1913)	67,240 (1974)
R_{work}/R_{free}[3]		21.2/24.9	21.5/27.9	20.3/24.1
R.m.s.d.				
	bond lengths (Å)	0.007	0.008	0.008
	bond angles (˚)	1.09	1.21	1.13
Average B-factors				
	protein/detergent/water	37/50/41	49/-/43	45/67/46
Ramachandran				
	favored/outliers (%)	95.8/0.3	94.0/0.3	94.9/0.7
Molprobity Clashscore		13.1	21.2	20.2

[1]Values in parentheses are for the highest resolution shell

[2]Values in parentheses are the number of reflections used to calculate R_{free}

[3]$R_{work} = \sum|Fo–Fc|/\sum Fo$. R_{free} is the cross-validation of the R-factor
