# Supplementary material for: Substrate Specificity within a Family of Outer Membrane Carboxylate Channels
Source: PLoS Biol. 2012 Jan 17;10(1):e1001242. doi: 10.1371/journal.pbio.1001242 (PMC3260308; doi:10.1371/journal.pbio.1001242)
Supplement: Table S3 — Definition of Occ channel specificity. Uptake of radiolabeled arginine by OccD1, benzoate by OccK1, and glucuronate by OccK2 was measured in the presence of a 100-fold excess of unlabeled low-molecular weight compound. In all cases, total levels of uptake are reported, expressed as a percentage of uptake in the absence of unlabeled compound (100%). Substrates containing a carboxyl group are italicized. (PDF) [file pbio.1001242.s018.pdf]

Table S3. Definition of Occ channel specificity. Uptake of radiolabeled arginine by OccD1, benzoate by OccK1, and glucuronate by OccK2 was measured in the presence of a 100-fold excess of unlabeled low-molecular weight compound[a]. In all cases, total levels of uptake are reported, expressed as a percentage of uptake in the absence of unlabeled compound (100%). Substrates containing a carboxyl group are italicized.

	OccD1	OccK1	OccK2
Control	100 ± 10	100 ± 6	100 ± 9
**Amino acids**			
Lysine/Arginine	**0 ± 6**[b]**/0 ± 5**	80 ± 7/84 ± 4	75 ± 2/81 ± 3
D-arginine/Histidine	**5 ± 1/0 ± 3**	90 ± 4/59 ± 8	89 ± 3/73 ± 6
Ornithine/Agmatine	**0 ± 5**/74 ± 3	89 ± 6/85 ± 5	86 ± 4/95 ± 2
Aspartic Acid/Proline	91 ± 5/92 ± 2	**20 ± 6**/55 ± 5	**21 ± 2**/50 ± 4
Leucine/Tyrosine	97 ± 3/90 ± 3	67 ± 4/64 ± 6	90 ± 3/85 ± 5
Serine/Pyroglutamate	91 ± 4/80 ± 2	**41 ± 2/1 ± 2**	87 ± 2/**5 ± 3**
Arg-Arg dipeptide	**3 ± 2**	85 ± 3	88 ± 3
**Sugars**			
Glucose/Sucrose	98 ± 5/90 ± 4	79 ± 3/74 ± 4	90 ± 5/78 ± 3
Galactose/Arabinose	79 ± 3/89 ± 8	74 ± 5/79 ± 5	63 ± 4/79 ± 4
Gluconate/Glucuronate	94 ± 3/89 ± 2	57 ± 8/**1 ± 5**	90 ± 3/**0 ± 2**
**Aromatic compounds**			
Benzoate/Vanillate	82 ± 3/90 ± 4	**0 + 3/9 ± 2**	**0 ± 3**/70 ± 4
3-nitrobenzoate	96 ± 5	**5 ± 8**	**37 ± 6**
4-nitrobenzoate	97 ± 2	**1 ± 4**	**25 ± 4**
**Organic Acids**			
Lactate/EDTA	77 ± 6/97 ± 1	92 ± 7/50 ± 4	95 ± 7/91 ± 8
Citrate/Cis-aconitate	95 ± 8/97 ± 4	92 ± 3/90 ± 5	95 ± 5/98 ± 2
Succinate/Malonate	78 ± 4/91 ± 3	98 ± 5/95 ± 5	79 ± 5/77 ± 3
Tartrate/Adipate	82 ± 5/92 ± 5	80 ± 4/**2 ± 2**	96 ± 2/93 ± 3
**Fatty acids/alcohols**			
Caproate/Octanoate	92 ± 4/96 ± 6	**1 ± 3/1 ± 6**	98 ± 6/91 ± 3
Octanol	91 ± 8	96 ± 5	97 ± 2
**Others**			
Adenosine/Thymidine	74 ± 2/87 ± 6	94 ± 8/96 ± 7	58 ± 6/76 ± 2
Indole/Imidazole	92 ± 5/75 ± 3	90 ± 8/67 ± 4	91 ± 1/93 ± 6

[a]Reported values are the average of two or three experiments.

[b]Efficient inhibition values, defined as resulting in <50% transport, are shown in bold.
