# Supplementary material for: Substrate Specificity within a Family of Outer Membrane Carboxylate Channels
Source: PLoS Biol. 2012 Jan 17;10(1):e1001242. doi: 10.1371/journal.pbio.1001242 (PMC3260308; doi:10.1371/journal.pbio.1001242)
Supplement: Text S1 — Presentation and discussion of the revised nomenclature for Occ proteins and detailed descriptions of methods and a supporting reference. (PDF) [file pbio.1001242.s019.pdf]

Supporting Discussion

Revised nomenclature for the OprD channel family

The previous nomenclature for members of the Occ/OprD family was arbitrary because no transport substrate was known for any of these channels, with the exception of OprD (OprD stands for outer membrane protein D). Moreover, the nomenclature was very confusing due to the fact that several unrelated channels have very similar names. For example, OprP is a trimeric, specific phosphate channel of *P. aeruginosa* that is completely unrelated in structure and sequence to OprD channels, including OpdP. Another important *P. aeruginosa* channel protein is OprB, which is a saccharide-specific channel of unknown structure. Again, this protein has no sequence similarity to any OprD family channel, including OpdB.

In this manuscript we show that OprD channels transport substrates with a carboxyl group. This, combined with the confusing existing nomenclature, is the basis for our proposal to rename this family Occ, for outer membrane carboxylate channels. In the new nomenclature (Table S1), the archetypes of the two subfamilies are OccD1 (formerly OprD) and OccK1 (formerly OpdK). Those family members for which we have obtained structures and biophysical/biochemical data have the lowest numbers. A clear advantage of the new nomenclature is that it is now obvious to which subfamily each member belongs (see Table S1).

Supporting Methods

Preparation of Proteoliposomes

P. putida cells were grown overnight at 25ºC. Cells were harvested by centrifugation at 4500 rpm for 10 min (Beckman Coulter, J6-MC). Cell pellets were suspended in deionized H_2O and centrifuged at 5000 rpm for 5 min. Cell pellets were resuspended in 2:1 chloroform:methanol mixture and incubated at 25ºC for 20 min on a rocking shaker. The suspension was filtered through Whatman filter #1 (GE Healthcare, Piscataway,

NJ). 0.9% NaCl was added to the filtrate to a final concentration of 0.18% and vortexed vigorously. The phases were separated by centrifugation at 5000 rpm for 10 min. After the removal of the aqueous phase and the solution interface, chloroform was evaporated under nitrogen gas and the lipid film was dried. Lipids were hydrated by 10 mM Hepes, 50 mM KCl, pH 7.0 to a concentration of 20 mg/ml, and sonicated with a bath sonicator (Model G112SPIT, Laboratory Supplies Company, Hicksville, NY) for 30 m. After 3 freeze-thaw cycles, the liposomes were extruded (Avanti mini extruder, Avanti Polar Lipids, Alabaster, AL) using 0.4 micron nuclepore track-etch membrane filters (Whatman). Purified OccD1 was added to the liposomes to a 1:100 (w/w) ratio in the presence of 0.02% Triton X-100 and proteoliposomes were prepared by incubation at 25ºC for 30 min on a rocking shaker. In order to remove Triton X-100, proteoliposomes were incubated with biobeads (Bio-Rad, Hercules, CA) overnight at 4ºC. Biobeads were removed by centrifugation at 4000 rpm (Eppendorf Centrifuge 5417C) for 5 min. Non-incorporated proteins were removed by size exclusion chromatography.

Radiolabeled Arginine Uptake into Proteoliposomes

Proteoliposomes were diluted to 5 mg/ml in 10 mM HEPES, 50 mM KCl, pH 7.0 buffer. ^{3}H-arginine (PerkinElmer, Boston, MA) was added to 100 µl of proteoliposomes to a final concentration of 0.25 µM and the assay mixture was incubated at 25ºC for 15 min. Proteoliposomes were filtered using 0.22 micron nitrocellulose filters (Millipore, Billerica, MA) and washed with 3 ml of HEPES buffer. The filters were then placed in scintillation vials containing 5 ml Econo-Safe scintillation fluid (Atlantic Nuclear Corp., Rockland, MA) and counted using a LS 6500 multi-purpose scintillation counter (Beckman Coulter, Brea, CA).

Cloning, Expression and Purification of LAO-Binding Protein

The gene expressing periplasmic LAO-binding protein from *E. coli* was cloned into the *E. coli* expression vector pB22 with an N-terminal hexa-histidine tag for purification (PB22-LAO-BP). DNA sequencing was performed at CFAR DNA sequencing facility (UMass Medical School, Worcester, MA). BL21(DE3) T1 phage-resistant cells (New England Biolabs, Ipswich, MA) were transformed with pB22-LAO-BP The cells were grown to OD_{600} ~ 0.6 at 37ºC and then induced with 0.1% arabinose at 20ºC overnight. Cells were harvested by centrifugation at 4500 rpm for 30 min (Beckman Coulter, J6-MC). Cell pellets were suspended in TSB (20 mM Tris, 300 mM NaCl, 10% glycerol, pH 8.0) and cells were lysed by sonication (3 x 40 s intervals) (Branson Digital Sonifier). Periplasmic extract was obtained by centrifugation at 40,000 rpm for 40 min (45 Ti rotor, Beckman L8-70M ultracentrifuge). The extract was applied to a 10 ml nickel column. The column was washed with 10 column volumes (CV) TSB containing 15 mM imidazole. The proteins were eluted with 3 CV TSB containing 200 mM imidazole. The proteins were further purified by gel filtration chromatography using 10 mM Tris, 50 mM NaCl, pH 8.0. The purified protein was concentrated to 5-15 mg/ml using 10 kDa molecular weight cutoff filters (Amicon) and directly flash-frozen in liquid nitrogen, without dialysis.

Incorporation of LAO-Binding Protein into Proteoliposomes

P. putida liposomes were prepared as described above. In order to prepare OccD1 liposomes with internal LAO-binding protein, both purified OccD1 and purified LAO-binding protein were added simultaneously to the liposomes to a 1:100 (w/w) ratio in the presence of 0.02% Triton X-100 and proteoliposomes were prepared by incubation at 25ºC for 30 min on a rocking shaker. In order to remove Triton X-100, proteoliposomes were incubated with biobeads (Bio-Rad, Hercules, CA) overnight at 4ºC. Biobeads were removed by centrifugation at 4000 rpm (Eppendorf Centrifuge 5417C) for 5 min. The proteoliposomes were then subjected to an additional freeze-thaw cycle to ensure the internalization of LAO-binding protein followed by extrusion as described above. Unincorporated proteins were removed by size exclusion chromatography.

Optimization of Substrate Concentration and Time Courses for Radiolabeled Substrate Uptake in Membrane Vesicles

Membrane vesicles of selected Occ channels and *E. coli* OmpG were diluted to contain 1 mg/ml total membrane protein. Varying concentrations of each radiolabeled substrate (0.05 µM-10µM, see Figure S8) were added to 100 µl of diluted membrane vesicles and the assay mixture was incubated at 25ºC (uptake times for each substrate is indicated in the figure legend). For OccD1, samples were taken at various time points (Figure S9). The vesicles were filtered and washed (10 mM HEPES, pH 7.0) as described above. The filters were placed in scintillation vials containing 5 ml Econo-Safe scintillation fluid and counted using a LS 6500 multi-purpose scintillation counter.

Determination of Protein Orientation in Membrane Vesicles

Orientation of Occ channels in membrane vesicles was determined by detection of N-terminus histidine tag by dot-blot analysis of unboiled membrane vesicles. In right-side-out vesicles, the N-terminus should be pointing inside the vesicles and therefore would not be accessible to the antibody. Samples from membrane vesicles were collected after various freeze-thaw cycles (1-3) and diluted membrane vesicles were applied on nitrocellulose membranes. N-terminus histidine tag was detected using Penta-His HRP conjugate (Qiagen, Germantown, MD) and the band intensities were compared by protein-expressing vesicles solubilized with detergent (2% DM).

Effect of Polymyxin B on Arginine Transport into Vesicles

Arginine uptake into OccD1 membrane vesicles was carried out as described in the main text for 15 min. Subsequently, Polymyxin B (Sigma; 8610 units/mg) from a 10 % stock solution in 10 mM HEPES, pH 7.0 was added to a final concentration of 0.01%-0.5% (w/v) into the assay. After brief vortexing, the assay mixture was incubated for an additional minute at room temperature. Membrane vesicles were filtered, washed and analyzed as described in the main text. The amount of vesicles remaining on the

nitrocellulose filters were quantified as follows: 10 % polymyxin B was added to 100 µl of OccD1 vesicles (containing 1 mg/ml total membrane) to a final concentration of 0.5% (the highest concentration used to permeabilize vesicles). After filtering the vesicles and washing, the filters were placed in 1.5 ml microcentrifuge tubes and the total protein remaining on the filters was quantified using the BCA assay.

Supporting Reference

1- Smart OS, Goodfellow JM, Wallace BA (1993). The pore dimensions of Gramicidin A. Biophys. J. 65: 2455-2460.
